# Induction of salivary antibody levels in Dutch adolescents after immunization with monovalent meningococcal serogroup C or quadrivalent meningococcal serogroup A, C, W and Y conjugate vaccine

**DOI:** 10.1371/journal.pone.0191261

**Published:** 2018-04-19

**Authors:** Mariëtte B. van Ravenhorst, Gerco den Hartog, Fiona R. M. van der Klis, Debbie M. van Rooijen, Elisabeth A. M. Sanders, Guy A. M. Berbers

**Affiliations:** 1 CIb, National Institute of Public Health and the Environment (RIVM), Bilthoven, The Netherlands; 2 Department of Paediatric Immunology and Infectious Diseases, Wilhelmina Children’s Hospital, University Medical Center, Utrecht, The Netherlands; Public Health England, UNITED KINGDOM

## Abstract

**Background:**

Meningococcal infection starts with colonisation of the upper respiratory tract. Mucosal immunity is important for protection against acquisition and subsequent meningococcal carriage. In this study, we assessed salivary antibody levels against meningococcal serogroup A (MenA), W (MenW) and Y (MenY) after vaccination with a quadrivalent MenACWY conjugated vaccine. We also compared salivary meningococcal serogroup C (MenC) antibody levels after monovalent MenC and quadrivalent MenACWY conjugated vaccination.

**Methods:**

Healthy participants, who had received MenC conjugate vaccine between 14 months and 3 years of age, received a (booster) MenC or MenACWY vaccination at age 10–15 years. MenA-, MenC-, MenW- and MenY-polysaccharide (PS) specific IgG and IgA levels in saliva and serum and PS specific secretory component levels in saliva were measured using the fluorescent-bead-based multiplex immunoassay.

**Results:**

MenACYW vaccination increased salivary PS-specific IgA (2-fold) and IgG levels(>10-fold) for MenA, MenY, and MenW. After one year, salivary IgA levels had returned to baseline levels. Both vaccines induced an increase in salivary MenC-PS specific IgA (>3-fold) and IgG (>100-fold), with higher levels after MenC as compared to MenACWY vaccination. The antibody decay rate of MenC in saliva between one month and one year was similar for both vaccines. The overall correlation between serum and saliva IgA levels was low (R = 0.39, R = 0.58, R = 0.31, and R = 0.36 for MenA, MenC, MenW and MenY, respectively). Serogroup-PS specific IgG levels between serum and saliva correlated better (R ranged from 0.51 to 0.88).

**Conclusions:**

Both primary (MenA, MenY, and MenW) and booster (MenC) parenteral meningococcal conjugate vaccination induced high salivary antibody levels. The strong correlation for MenC, MenW and MenY between saliva and serum IgG levels indicates that saliva might be used as a reliable tool to measure vaccine responses after both primary and booster meningococcal vaccination.

## Introduction

Invasive meningococcal disease (IMD), caused by *Neisseria meningitidis*, is a devastating disease and associated with substantial mortality and morbidity [1]. Transmission occurs through air-borne droplets or direct contact with respiratory secretion. Peak incidence of IMD is observed in children below the age of five years, with a second smaller peak of incidence in adolescents [2]. Based on the polysaccharide capsule, the main virulence factor, twelve serogroups can be classified of which six (A, B, C, W, X and Y) are the dominant disease-associated serogroups.

Meningococcal conjugate vaccines (MCVs) induce systemic polysaccharide (PS) specific IgG levels that are important for individual protection against IMD. Besides individual protection, MCVs establish herd protection by reducing meningococcal acquisition and transmission of meningococci to the unprotected population [3–6]. Adolescent vaccination, the age group with the highest carriage rates [7–9], is now considered to play a key role in protection of the population against IMD by herd protection.

In response to a steep increase of MenC IMD cases at the end of the 20^th^ century in the Netherlands, a single MenC conjugated (MenCC) vaccine was offered to all children aged 1–18 years during a large catch-up campaign in 2002 with vaccination coverage of 94% [10]. At the same time, a routine MenCC vaccination at 14 months of age was implemented in the Dutch immunization program (NIP). MenC antibody levels however wane considerably after infant immunization with MenCC vaccine leaving older children again unprotected [11]. For this reason, adolescent booster vaccination was introduced in countries like the UK not only to boost individual protection but also to sustain herd protection in the population. After virtual disappearance of MenC IMD, rapid increases of meningococcal serogroup W (MenW) disease are observed recently in several European countries including the Netherlands [12, 13]. For this reason, the UK switched from monovalent MenCC to quadrivalent MenACWY conjugate vaccination in adolescents [14]. Several other countries also consider MenACWY vaccination for adolescents.

To achieve herd protection, local immunity is required to protect against acquisition, colonization, and spread of the meningococcus. Salivary antibodies are thought to play an important role in establishing herd protection by prevention attachment of microbes to the epithelium and by promoting elimination from mucosal surfaces [15, 16]. This process is also referred to as immune-exclusion and has been described for mucosal IgA. We previously demonstrated that both monovalent and quadrivalent MCVs elicited robust serum immune responses against all targeted serogroups in adolescents who had been primed with a single MenCC vaccination [17, 18]. To our knowledge, no previous studies assessed salivary antibody response against all targeted serogroups following adolescent quadrivalent MenACWY vaccination in an European adolescent population.

In this study, we assessed primary salivary antibody levels against MenA, MenW and MenY and compared booster MenC salivary levels between monovalent MenC-TT and quadrivalent MenACWY-TT vaccination. Furthermore, salivary antibody levels were compared with serum antibody levels of the corresponding serogroup.

## Methods

### Ethics statement

This study was designed and conducted in accordance with Good Clinical Practice guidelines established by the International Conference on Harmonization and with the Declaration of Helsinki. Ethical approval was obtained from the local Medical research Ethics Committees United (MEC-U). Written informed consent was obtained from both parents or guardians and subjects aged ≥12 years before enrollment. This study was registered at the EU Clinical Trials database (EudraCT number: 2013-001823-38) and at the Dutch Trial Register (www.trialregister.nl; NTR4430).

### Study design and clinical procedures

This study was part of a phase IV, single center, open-label controlled trial to demonstrate non-inferiority of the MenACWY-TT vaccine compared to the MenC-TT vaccine. Detailed information on study design, recruitment, inclusion and exclusion criteria were previously described [17, 18]. In short, healthy adolescents immunized previously with a single MenC conjugated to tetanus toxoid (MenC-TT, NeisVac-C^TM^, Pfizer) vaccine between 14 months and 3 years of age, were included. One 0.5 mL dose of quadrivalent vaccine conjugated to tetanus toxoid (MenACWY-TT, Nimenrix^TM^, Pfizer) or one 0.5 mL dose of the MenC-TT vaccine was administered at age 10-, 12- or 15-years. Blood and saliva samples were collected before (T0), 1 month (T1) and 1 year (T2) after the adolescent vaccination. Saliva samples were collected using the Oracol Saliva Collection system (Malvern Medical Developments Limited). Participants were instructed to allow absorption of saliva into the swab for 1 minute. Saliva was immediately squeezed out the swab into a 2 mL spray-dried EDTA-tube (BD), and stored at ambient temperature. Within 24 hours after collection, saliva samples were centrifuged and stored at -80°C.

### Laboratory analyses

MenA-, MenC-, MenW- and MenY-PS specific IgG and IgA levels in serum and saliva and PS specific secretory component (SC) levels in saliva were measured using the fluorescent-bead-based multiplex immunoassay (MIA) as previously described [19–21]. All samples were tested in duplicate in the same run.

### Statistical analyses

All statistical analyses were performed in the according to protocol (ATP) cohort as previously described [17].To ensure that the results reliably reflect the antibody kinetics and the relation between saliva and serum, only participants with sufficient amount of saliva and serum to perform all isotype-specific measurements per serogroup at both one month (T1) and one year (T2) after the vaccination were included in the statistical analyses. Serum and salivary antibody concentrations were log transformed and presented as geometric mean concentrations (GMCs) with their 2-sided corresponding 95% confidence intervals (CI). Difference in antibody concentrations between the vaccine groups were analyzed with linear regression analyses and adjusted for baseline concentrations for all age groups together. Correlations were analyzed using Pearson’s correlation. Principal component analysis (PCA) was applied to reduce the data dimensionality without loss of information to assess similarity and differences between samples antibody levels. A p-value below 0.05 was considered statistically significant. Data were analyzed using SPSS statistics 22 (IBM) and GraphPad Prism 7.00.

## Result

### Participants

The original ATP cohort included 464 participants as previously described: 225/464 (48.5%) and 239/464 (51.5%) received the MenACWY-TT and MenC-TT vaccine, respectively [17, 18]. For MenC antibody levels in both serum and saliva at 1 month and 1 year of 421 participants (201 and 220 for MenACWY-TT and MenC-TT vaccine, respectively) were available, whereas 201 were available for MenA, and 202 for MenW and MenY (S1 Fig).

### MenA-, MenW- and MenY-PS-specific primary IgA responses

At baseline, serogroup-PS specific IgA levels in saliva and serum were low in both serum and saliva (Table 1A). The serum/saliva IgA ratio was similar between the serogroups (1.5, 1.2, 1.1 for MenA, MenW and MenY, respectively). In saliva, a 2-fold increase in IgA levels was measured one month post vaccination (T1/T0) for all three serogroups. In serum, increase in IgA ranged between 23-fold for MenA to 64-fold for MenY. In serum, higher IgA antibody decay between one month and one year (T1/T2) was observed for MenA (4.3-fold) compared to MenW (2.2-fold) and MenY (2.5-fold), whereas salivary IgA antibody decay was similar between the three serogroups. IgA levels in saliva one year after the primary vaccination were comparable to levels at baseline. In serum however, IgA levels were still 5-fold higher than levels at baseline for MenA, and 26-fold higher for MenW and MenY.

**Table 1:** Geometric mean concentration (GMC) of meningococcal serogroup A-, W- and Y-polysaccharide specific IgA (A) and IgG (B) in saliva and serum before (T0), one month (T1) and one year (T2) after adolescent vaccination.

			MenA	MenW	MenY
**A**	**T0**	**GMC IgA in saliva; ng/ml (95%CI)**	42 (37–46)	13.2 (12.0–14.5)	27.5 (25.0–30.2)
		**GMC IgA in serum; ng/ml (95%CI)**	61 (52–72)	15.6 (13.0–18.8)	31 (25–38)
	**T1**	**GMC IgA in saliva; ng/ml (95%CI**	88 (79–99)	27 (24–31)	52 (47–59)
		**T1/T0 IgA ratio saliva (95%CI)**	2.1 (1.8–2.5)	2.1 (1.8–2.4)	1.9 (1.7–2.2)
		**GMC IgA in serum; ng/ml (95%CI**	1,427 (1,225–1,662)	877 (736–1,045)	1,985 (1,658–2,376)
		**T1/T0 IgA ratio serum (95%CI)**	23 (18–28)	58 (46–73)	64 (50–82)
	**T2**	**GMC IgA in saliva; ng/ml (95%CI**	33 (29–37)	10.7 (9.4–12.3)	25 (23–28)
		**T2/T0 IgA ratio saliva (95%CI)**	0.80 (0.71–0.90)	0.81 (0.71–0.93)	0.93 (0.84–1.02)
		**T1/T2 IgA ratio saliva (95%CI)**	2.7 (2.4–3.0)	2.5 (2.2–3.0)	2.1 (1.8–2.3)
		**GMC IgA in serum; ng/ml (95%CI**	330 (282–386)	403 (350–464)	797 (662–960)
		**T2/T0 IgA ratio serum (95%CI)**	5.35 (4.39–6.50)	26.12 (21.35–31.98)	25.80 (20.32–32.78)
		**T1/T2 IgA ratio serum (95%CI)**	4.3 (3.8–4.9)	2.2 (1.9–2.5)	2.5 (2.2–2.8)
**B**	**T0**	**GMC IgG in saliva; ng/ml (95%CI)**	4.0 (3.0–5.4)	0.2 (0.1–0.2)	0.4 (0.3–0.5)
		**GMC IgG in serum; ng/ml (95%CI)**	540 (462–624)	119 (97–146)	65 (54–77)
	**T1**	**GMC IgG in saliva; ng/ml (95%CI**	38.4 (31.2–47.3)	4.6 (3.8–5.7)	4.8 (3.9–5.8)
		**T1/T0 IgG ratio saliva (95%CI)**	10 (6.9–13.3)	28 (21–38)	12 (9.5–15.0)
		**GMC IgG in serum; ng/ml (95%CI**	27,766 (23,575–32,701)	5,051 (4,104–6,217)	6,045 (4,967–7,358)
		**T1/T0 IgG ratio serum (95%CI)**	51 (43–61)	43 (33–54)	94 (76–115)
	**T2**	**GMC IgG in saliva; ng/ml (95%CI**	5.5 (4.1–7.3)	1.9 (1.4–2.4)	1.7 (1.4–2.1)
		**T2/T0 IgG ratio saliva (95%CI)**	1.35 (0.96–1.91)	11.35 (8.31–15.51)	4.24 (3.46–5.18)
		**T1/T2 IgG ratio saliva (95%CI)**	7.3 (5.5–9.7)	2.6 (2.0–3.3)	2.1 (1.8–2.3)
		**GMC IgG in serum; ng/ml (95%CI**	6,659 (5,778–7,673)	3,204 (2,692–3,813)	1,749 (1,417–2,160)
		**T2/T0 IgG ratio serum (95%CI)**	12.35 (10.89–14.01)	26.96 (21.38–34.01)	27.08 (22.16–33.09)
		**T1/T2 IgG ratio serum (95%CI)**	4.2 (3.6–4.8)	1.6 (1.3–1.9)	2.5 (2.2–2.8)

### MenA-, MenW- and MenY-PS-specific primary IgG responses

Serum IgG levels in this study of a subset of participants were similar to serum IgG levels for all participants which we previously described [18]. At baseline, salivary IgG levels were 135, 595, and 163 times lower than serum IgG levels for MenA, MenW and MenY, respectively (Table 1B). Similar to IgA, the highest IgG levels in serum and saliva at baseline were observed for MenA. One month after vaccination, a more than 10-fold increase in salivary IgG levels was observed, with the highest increase for MenW. The highest increase of serum IgG levels was observed for MenY (94-fold) compared to MenA (51-fold) and MenW (43-fold). The highest antibody decay in both salivary and serum IgG levels occurred between one month and one year (T1/T2) for MenA, but the absolute antibody levels remained higher than those for MenW and MenY. One year after the vaccination, both salivary and serum IgG levels were significantly higher for all three serogroups compared to baseline levels, except for MenA-PS specific IgG levels in saliva (Table 1B).

### Comparison of MenC-PS-specific booster IgA responses between vaccine groups

Already prior to administration of the booster vaccine, higher salivary IgA anti-MenC-PS antibody levels were observed in the MenACWY-TT vaccine group compared to the MenC-TT vaccine group (p-value <0.001; Table 2A). Serum IgA levels against MenC-PS were similar between vaccine groups (p-value 0.584).

**Table 2:** Geometric mean concentration (GMC) of meningococcal serogroup C-polysaccharide (MenC-PS) specific IgA (A) and IgG (B) in saliva and serum before (T0), one month (T1) and one year (T2) after adolescent vaccination.

			MenACWY-TT	MenC-TT	*p*-value
**A**	**T0**	**GMC MenC-PS specific IgA in saliva; ng/ml (95% CI)**	10.4 (9.4–11.4)	6.1 (5.6–6.8)	**<0.001**
		**GMC MenC-PS specific IgA in serum; ng/ml (95% CI)**	24.1 (19.7–29.6)	22.6 (18.8–27.1)	0.584
	**T1**	**GMC MenC-PS specific IgA in saliva; ng/ml (95% CI)**	31 (26–35)	43 (36–51)	**<0.001**
		**T1/T0 IgA ratio saliva (95% CI)**	3.0 (2.5–3.5)	7.1 (5.9–8.4)	**<0.001**
		**GMC MenC-PS specific IgA in serum; ng/ml (95% CI)**	9,786 (8,355–11,462)	10,939 (9,523–12,564)	0.261
		**T1/T0 IgA ratio serum (95% CI)**	415 (335–514)	485 (411–573)	0.260
	**T2**	**GMC MenC-PS specific IgA in saliva; ng/ml (95% CI)**	11.0 (9.7–12.5)	12.8 (10.9–15.1)	**<0.001**
		**T2/T0 IgA ratio saliva (95% CI)**	1.06 (0.94–1.20)	2.10 (1.78–2.49)	**<0.001**
		**T1/T2 IgA ratio saliva (95% CI)**	2.8 (2.4–3.2)	3.3 (2.8–3.9)	0.099
		**GMC MenC-PS specific IgA in serum; ng/ml (95% CI)**	1,085 (913–1,290)	1,424 (1,214–1,670)	**0.005**
		**T2/T0 IgA ratio serum (95% CI)**	45.07 (36.78–55.23)	63.43 (53.19–75.64)	0.154
		**T1/T2 IgA ratio serum (95% CI)**	9.0 (8.1–10.0)	7.7 (7.1–8.3)	**0.017**
**B**	**T0**	**GMC MenC-PS specific IgG in saliva; ng/ml (95% CI)**	0.9 (0.8–1.1)	0.8 (0.6–0.9)	0.218
		**GMC MenC-PS specific IgG in serum; ng/ml (95% CI)**	238 (201–278)	287 (252–327)	**0.044**
	**T1**	**GMC MenC-PS specific IgG in saliva; ng/ml (95% CI)**	110 (90–133)	231 (197–270)	**<0.001**
		**T1/T0 IgG ratio saliva (95% CI)**	123 (97–155)	307 (250–378)	**<0.001**
		**GMC MenC-PS specific IgG in serum; ng/ml (95% CI)**	139,600 (122,343–159,291)	159,696 (146,091–174,569)	0.136
		**T1/T0 IgG ratio serum (95% CI)**	590 (481–723)	556 (482–641)	0.638
	**T2**	**GMC MenC-PS specific IgG in saliva; ng/ml (95% CI)**	7.6 (6.3–9.2)	12.1 (10.0–14.6)	**<0.001**
		**T2/T0 IgG ratio saliva (95% CI)**	8.48 (6.94–10.36)	16.01 (12.80–20.01)	**0.001**
		**T1/T2 IgG ratio saliva (95% CI)**	14.4 (11.8–17.6)	19.1 (15.8–23.2)	**0.046**
		**GMC MenC-PS specific IgG in serum; ng/ml (95% CI)**	10,877 (9,565–12,370)	15,836 (14,062–17,833)	**<0.001**
		**T2/T0 IgG ratio serum (95% CI)**	45.96 (38.85–54.39)	55.12 (47.76–63.60)	0.589
		**T1/T2 IgG ratio serum (95% CI)**	12.8 (11.3–14.6)	10.1 (9.1–11.2)	**0.004**

One month after the booster, a higher increase (T1/T0) in salivary MenC-PS IgA levels was observed in the MenC-TT vaccine group compared to the MenACWY-TT vaccine group (p-value <0.001), resulting in significantly higher salivary IgA MenC-PS levels in the MenC-TT vaccine group (p-value <0.001). Serum IgA MenC-PS levels between vaccine groups at that time point were similar (p-value 0.260), resulting in similar levels of increase (T1/T0) for serum IgA.

One year after the booster, the level of decline (T1/T2) in salivary IgA was similar between vaccine groups (p-value 0.099). Higher serum IgA MenC-PS levels were observed in the MenC-TT vaccine group (p-value 0.005), due to a higher antibody decay between 1 month and 1 year after the booster in the MenACWY-TT vaccine group (p-value 0.017). Salivary IgA MenC-PS levels were comparable to pre-booster levels in the MenACWY-TT vaccine group, whereas salivary IgA MenC-PS levels were 2-fold higher in the MenC-TT vaccine group. Serum IgA levels were still higher than pre-booster levels in both vaccine groups (45-fold and 63-fold for the MenACWY-TT and MenC-TT vaccine group, respectively).

### Comparison of MenC-PS-specific booster IgG responses between vaccine groups

The pre-booster levels of salivary MenC-PS IgG were similar between vaccine groups, but were significantly different for serum levels (p-value 0.218 and 0.044 for saliva and serum, respectively). As observed for salivary IgA MenC-PS levels, a higher level of increase (T1/T0) in salivary IgG levels and higher absolute salivary IgG levels one month after the booster were observed in the MenC-TT vaccine group compared to the MenACWY-TT vaccine group (both p-value <0.001). One month after the booster, similar MenC-PS specific IgG in serum were observed between vaccine groups. One year after the booster, both IgG levels in saliva and serum remained higher in the MenC-TT vaccine group (p-value <0.001 for both saliva and serum). In both vaccine groups, salivary and serum IgG levels remained higher than pre-booster levels (8- and 45-fold for the MenACWY-TT and 15- and 55-fold for MenC-TT vaccine groups, respectively). At all time points, much lower IgG MenC-PS levels were observed in saliva compared to serum.

### Correlation between salivary and serum antibody levels and secretory component

**Fig 1:**
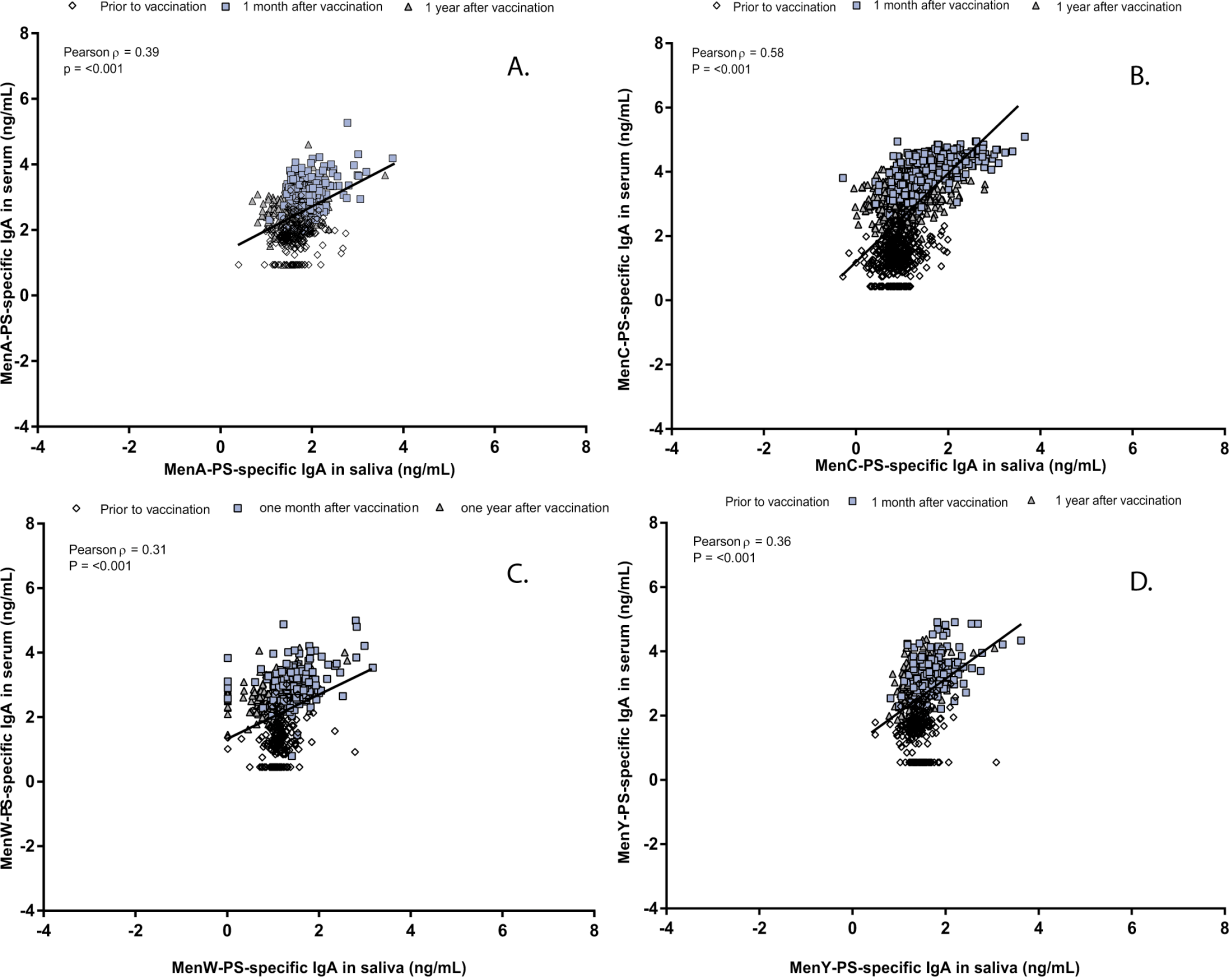
Correlation between log-transformed Immunoglobulin A (IgA) levels in saliva and serum for meningococcal serogroup A (A), C (B), W (C), and Y (D).

**Fig 2:**
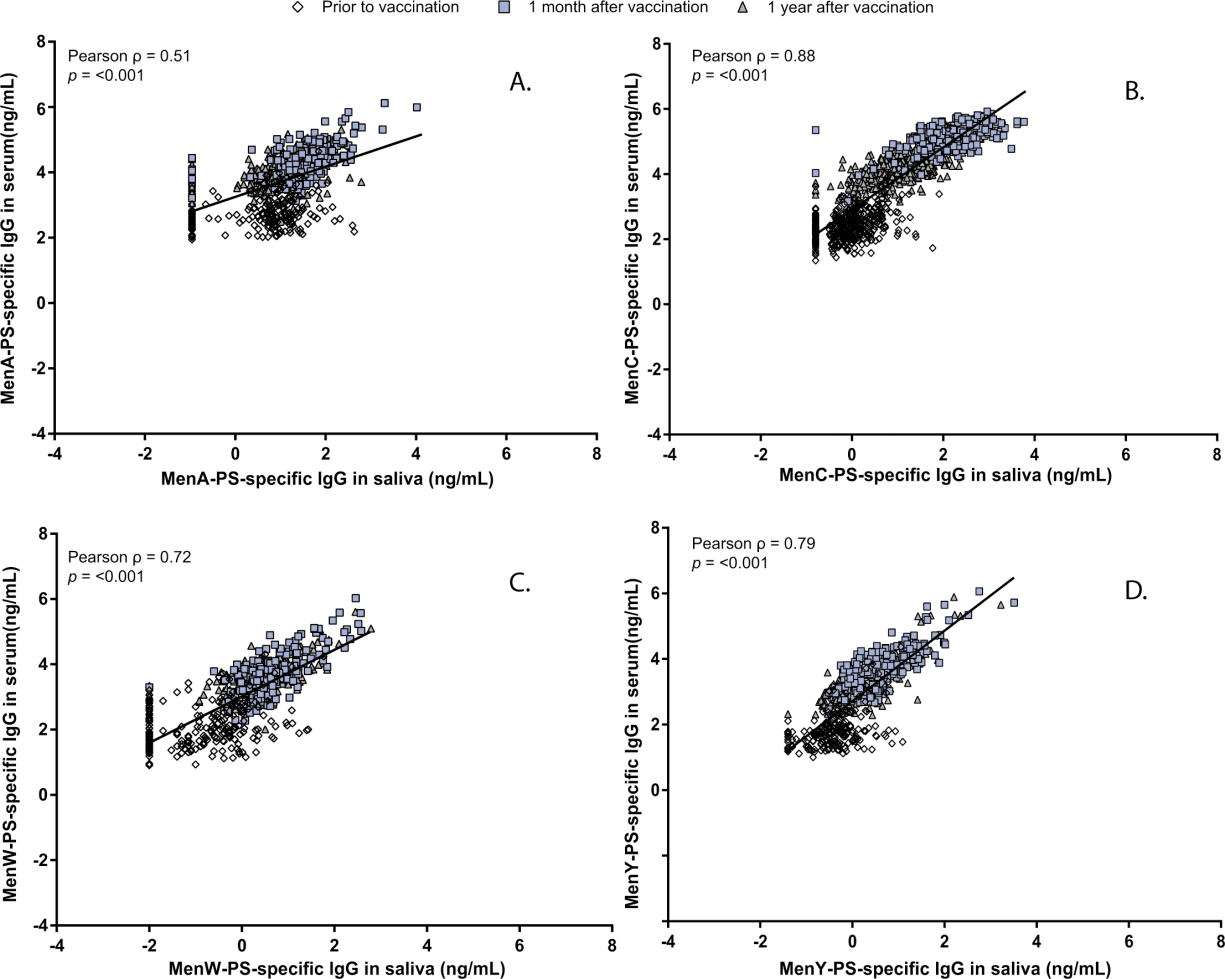
Correlation between log-transformed Immunoglobulin G (IgG) levels in saliva and serum for meningococcal serogroup A (A), C (B), W (C), and Y (D).

**Fig 3:**
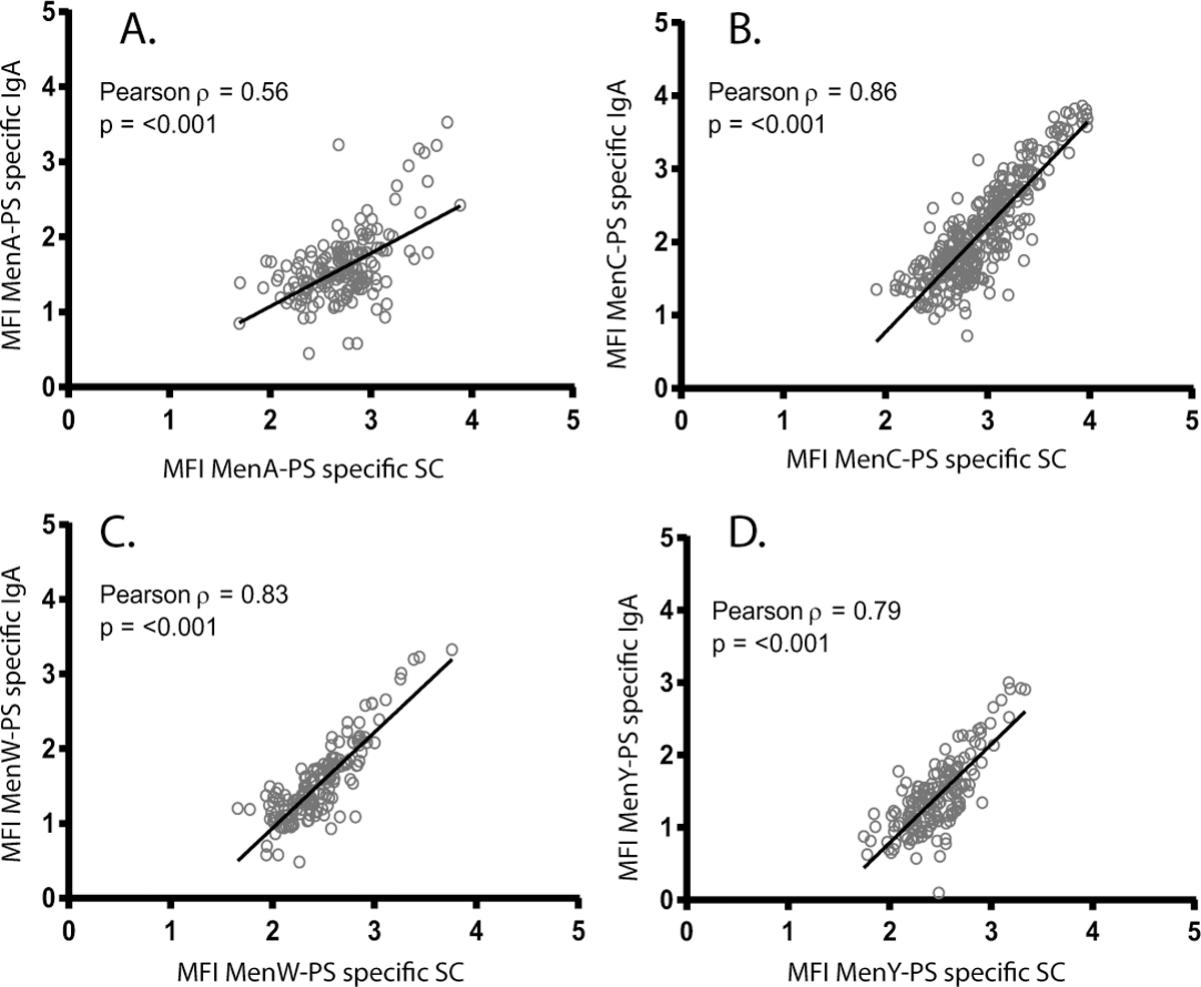
Correlation between log-transformed Immunoglobulin A (IgA) and secretory component (SC) fluorescent intensity levels (MFIs) in saliva for meningococcal serogroup A (A), C (B), W (C), and Y (D).

We observed comparable correlations between the two vaccine groups (data not shown), and therefore we combined the results for MenC for the two vaccines. The overall correlation between serum IgA and saliva IgA levels was found to be R = 0.39, R = 0.58, R = 0.31, and R = 0.36 for MenA, MenC, MenW and MenY, respectively (all p-values <0.001; Fig 1A–1D). Salivary IgG levels correlated better with the corresponding serum IgG levels for the four serogroups with coefficients varying from 0.51 for MenA to 0.88 for MenC (all p-values <0.001; Fig 2A–2D). Mean fluorescent intensity levels (MFIs) of IgA in saliva correlated significantly with the corresponding MFIs of the SC in saliva one month after the (booster) vaccination for all serogroups, with R = 0.56, R = 0.86, R = 0.83, and R = 0.79 for MenA, MenC, MenW and MenY, respectively (all p-values <0.001; Fig 3A–3D).

### Principal component analysis

**Fig 4:**
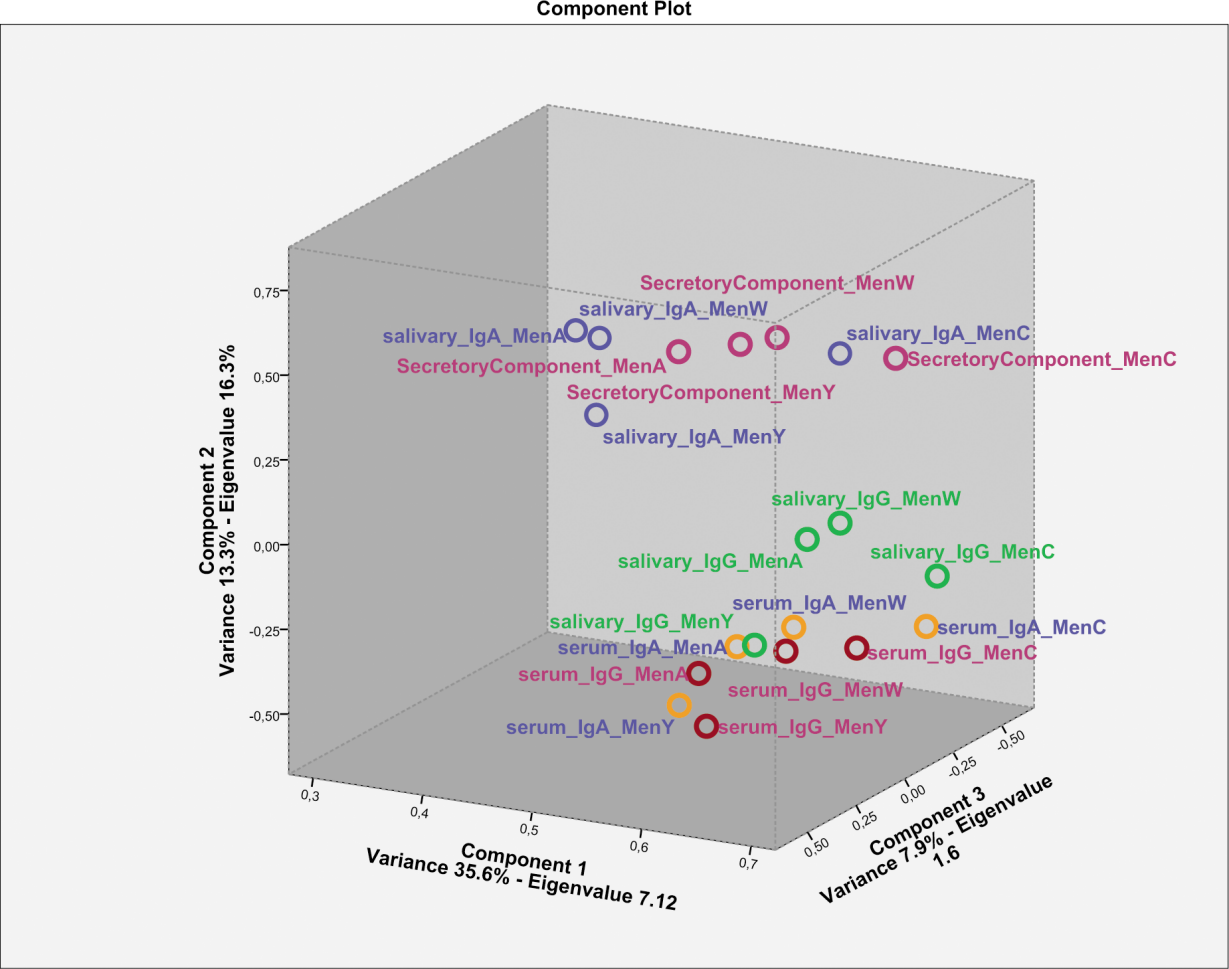
Principal components analysis of serum and saliva IgG and IgA levels to the different meningococcal serotypes. For this analysis, data of T1 was selected as these data had the highest variance. The Eigenvalues (have to exceed 1 to be explanatory) and the variance explained along the component axes are indicated. SBA = serum bactericidal assay.

Following our observation that the correlations between specific levels of IgG in serum and saliva were better than the correlation between IgA in serum and saliva, we analysed the data using principal component analysis. This analysis aimed to identify whether IgA and IgG levels in serum and saliva suggest to be induced from different origins. The first three components of the principal component analysis explain a significant amount of the variance (57%, Eigenvalues ≥1.8, Fig 4) and show that salivary IgG is similar to serum IgG, whilst salivary IgA clusters separately from serum IgA.

## Discussion

Salivary antibodies may play an important role in reducing acquisition and colonization of the targeted serogroups in vaccines after PS protein conjugate vaccinations, and thereby protect susceptible individuals in the population against invasive bacterial disease like IMD [22]. Both monovalent and quadrivalent meningococcal conjugate vaccines induce PS specific salivary IgG and IgA antibody production against all targeted serogroups. When comparing monovalent versus the quadrivalent MenC-PS containing conjugate vaccines higher MenC-PS specific IgA levels in saliva were found up to one year after monovalent vaccination. These individuals were primed in their first year of life with the monovalent MenC-TT vaccine. Serogroup-specific salivary IgG levels correlated significantly with the corresponding serum IgG levels while serum and saliva IgA levels correlated modestly but also significant for all targeted serogroups. Salivary IgA concentrations correlated highly with salivary SC levels for all four serogroups. For all analyses, MenA correlation was lower compared to the other three serogroups.

Previous studies showed that parental MenCC vaccines are able to induce salivary antibodies [19, 23, 24]. Information about salivary responses against MenW and MenY after quadrivalent vaccination is limited [25]. The current study showed that the MenACWY-TT vaccine in adolescents elicited salivary IgA and IgG responses against MenA, MenW and MenY. Salivary IgA, the main class of antibodies in saliva, might be important for herd protection by limiting meningococcal adherence to the oral surfaces [26, 27]. Although levels observed for IgG were much higher than IgA in serum for all four targeted serogroups, salivary IgA levels for MenA, MenW and MenY were higher than salivary IgG levels. However, we observed that salivary IgA levels at one year after both primary and booster vaccination dropped to pre-vaccination levels, indicating that the vaccines failed to induce long-lasting presence of specific IgA in saliva. Therefore, additional immune mechanisms may be important in limiting carriage and for herd protection. A previous study showed that macrophages, recruited to the mucosal surface by CD4 cells that express IL-17, might play an important role for clearing pneumococcal colonization in mice [28]. Interestingly, Mitsi et al. recently showed in a murine model that pneumococcal agglutination mediated by IgG to capsular PS specific antibodies is a key mechanism of protection against acquisition of carriage, leading to herd protection in the population [22]. Although the exact mechanism of herd protection after MCV administration is still unclear, it likely might also depend on agglutination mediated by meningococcal IgG antibodies in saliva. To define the exact mechanism for herd protection after vaccination with MCVs, further investigation is required.

Higher IgA and IgG absolute levels in saliva were observed for MenA at baseline compared to MenW and MenY. As no significant MenA carriage [7] and no MenA disease cases have been observed in the Netherlands, it is less likely to be caused by natural immunization. Though natural immunization cannot be excluded, antigens expressed by other bacteria which also can induce antibodies that crossreact with PS-specific MenA are more likely [29, 30]. We confirmed the specificity of our assay by performing blocking experiments in which we incubated a subset of serum and saliva samples collected at all three time points with homologous PS. After incubation, antibodies were still detected in baseline samples for MenA whereas MenW and MenY levels dropped under the lower limit of detection (data not shown). These results suggest that the MenA-PS antibodies at baseline may be cross-reactive antibodies originating from other species.

It has to be noted that we observed higher MenC pre-booster IgA levels in the quadrivalent vaccine group than in the monovalent vaccine group, which we cannot explain since the participants were randomized. After correction for pre-booster IgA levels MenC-specific mucosal IgA levels after monovalent vaccination were still significantly higher than after quadrivalent vaccination. The MenC-TT vaccine contains twice the amount of MenC PS compared to the MenACWY-TT vaccine (10µg vs 5µg, respectively), which may explain the higher salivary IgA and IgG levels in the monovalent vaccine group.

The strong correlation between salivary and serum IgG levels found in our study for MenC, MenW and MenY supports the current view that salivary IgG is mostly serum-derived [24, 31, 32]. This good correlation between saliva and serum IgG at one month and one year suggests that salivary samples might be a useful tool to screen IgG responses after both primary MenW and MenY) and booster (MenC) meningococcal vaccination for at least up to one year. However, the correlation between salivary and serum IgG levels for MenA was lower than for MenC, MenW and MenY. As previously described, cross-reactive antibodies might explain this lower correlation [29]. In addition, a better correlation was observed after booster response (MenC) compared to primary responses (MenW and MenY). This might be due to the induction of other immunoglobulins rather than IgG (ie. IgM) after primary vaccination. Nevertheless, evaluation of salivary antibodies after parenteral meningococcal immunization might reflect a surrogate of protection, rather than a correlate of protection but certainly warrants further exploring.

Salivary antibodies can be of systemic origin and thereby reflect levels observed in serum, or can be produced locally in mucosal tissues resulting in a lesser correlation with antibodies in serum. Our findings are in line with recent studies reporting significant correlations between IgA levels in saliva and serum [19, 25]. However, we found lower correlation compared to those studies. This might be explained by several factors that influence IgA levels in saliva (ie. age, carriage and the use of different vaccines [26]) hampering the comparison between the results of this study and previous findings. Nevertheless, the significant correlation between IgA levels in serum and saliva in all studies suggest that salivary IgA levels might also depend on the systemic response after parental vaccination. In contrast to central immunological tissues, mucosal production of antibodies is biased towards dimeric IgA. In contrast to monomeric IgA, the J chain of dimeric IgA is capable of binding to the polymeric immunoglobulin receptor on the epithelium and thereby facilitates its transport over the epithelium resulting in SC-bound dimeric IgA, also referred to as secretory IgA. Compared to IgG, correlation coefficients between salivary and serum IgA were lower whilst strong correlations between salivary IgA and SC were found. In addition to the observation that the ratio between serum IgG and salivary IgG was higher than the ratio between serum IgA and salivary IgA, it is likely that the majority of IgA is produced locally and mainly depends on the IgA trans-epithelial transport using the polymeric Ig receptor that results in SC-bound secretory IgA [24, 31, 32]. This was confirmed by the principal components analysis which showed a clear separation between salivary IgA and salivary SC from serum IgG, serum IgA and salivary IgG. The observation that salivary IgA correlates with epithelial-derived secretory component, that only binds to locally produced dimeric IgA (or IgM)[33, 34], confirms that the majority of salivary IgA was produced locally. Due to this strong correlation between salivary IgA and SC, it may be assumed that only a small proportion of the PS-specific B cells migrated to mucosal effector tissues. This reflects the better efficiency of the vaccines to induce central memory IgG cells compared to the induction of mucosal-homing IgA B cells.

Collection of saliva has major advantages compared to collection of serum: it is a non-invasive and painless method with limited costs especially useful in large-scale surveillance studies. In addition, saliva is easy to collect which is beneficial particular in geographical area where blood collection is difficult. Despite the use of standardized collection methods, differences in individual secretory flow rate and protein loss during sampling between and within individuals might hinder interpretation of the results [35]. In order to correct for individual variation in secretary flow rate, we analyzed salivary antibody levels also as fractions of the total protein concentration in saliva or total IgA levels. In accordance with our previous findings, similar results were observed after correction for total protein concentration (data not shown), suggesting that the current method (without correction) is accurate for quantification of individual salivary levels [19].

In conclusion, both primary and booster parenteral meningococcal conjugate vaccination induced antibody levels in saliva for all targeted serogroups. Salivary samples might be an interesting screening tool to measure vaccine responses after both primary and booster vaccination, especially in geographical areas where blood collection is challenging. While a minimal threshold needs yet to be assessed, IgG antibodies in saliva samples potentially serve as surrogate of protection.

## Supporting information

S1 Fig Flow diagram for follow up. Participants received a single vaccination with the Meningococcal serogroup C conjugated to tetanus toxoid (MenC-TT) vaccine or a quadrivalent Meningococcal serogroup A, C, W and Y conjugated to tetanus toxoid (MenACWY-TT) vaccine at enrolment. Blood abd saliva samples were collected before and 1 month and 1 year after this vaccination. ATP = According To Protocol.
(DOC)

S1 Table Correlation between serum and saliva serogroup-specific IgA and IgG concentrations per time point. (DOCX)

S1 File Study protocol. (PDF)

S2 File TREND checklist. (DOCX)

## References

[1] Harrison LH, Trotter CL, Ramsay ME. Global epidemiology of meningococcal disease. Vaccine. 2009;27 Suppl 2:B51–63. doi: 10.1016/j.vaccine.2009.04.063 . 19477562 10.1016/j.vaccine.2009.04.063

[2] Bijlsma MW, Bekker V, Brouwer MC, Spanjaard L, van de Beek D, van der Ende A. Epidemiology of invasive meningococcal disease in the Netherlands, 1960–2012: an analysis of national surveillance data. The Lancet Infectious diseases. 2014;14(9):805–12. Epub 2014/08/12. doi: 10.1016/S1473-3099(14)70806-0 . 25104306 10.1016/S1473-3099(14)70806-0

[3] Bijlsma MW, Brouwer MC, Spanjaard L, van de Beek D, van der Ende A. A decade of herd protection after introduction of meningococcal serogroup C conjugate vaccination. Clinical infectious diseases: an official publication of the Infectious Diseases Society of America. 2014;59(9):1216–21. Epub 2014/07/30. doi: 10.1093/cid/ciu601 . 25069869 10.1093/cid/ciu601

[4] Campbell H, Andrews N, Borrow R, Trotter C, Miller E. Updated postlicensure surveillance of the meningococcal C conjugate vaccine in England and Wales: effectiveness, validation of serological correlates of protection, and modeling predictions of the duration of herd immunity. Clinical and vaccine immunology: CVI. 2010;17(5):840–7. doi: 10.1128/CVI.00529-09 ; PubMed Central PMCID: PMC2863391. 20219881 10.1128/CVI.00529-09 PMC2863391

[5] Maiden MC, Ibarz-Pavon AB, Urwin R, Gray SJ, Andrews NJ, Clarke SC, et al Impact of meningococcal serogroup C conjugate vaccines on carriage and herd immunity. The Journal of infectious diseases. 2008;197(5):737–43. Epub 2008/02/15. doi: 10.1086/527401 . 18271745 10.1086/527401 PMC6767871

[6] Ramsay ME, Andrews NJ, Trotter CL, Kaczmarski EB, Miller E. Herd immunity from meningococcal serogroup C conjugate vaccination in England: database analysis. Bmj. 2003;326(7385):365–6. Epub 2003/02/15. ; PubMed Central PMCID: PMCPmc148893. 12586669 10.1136/bmj.326.7385.365 PMC148893

[7] Ravenhorst MB, Bijlsma MW, van Houten MA, Struben VM, Anderson AS, Eiden J, et al Meningococcal carriage in Dutch adolescents and young adults; A cross-sectional and longitudinal cohort study. Clinical microbiology and infection: the official publication of the European Society of Clinical Microbiology and Infectious Diseases. 2017 Epub 2017/02/14. doi: 10.1016/j.cmi.2017.02.008 . 28192234 10.1016/j.cmi.2017.02.008

[8] Christensen H, May M, Bowen L, Hickman M, Trotter CL. Meningococcal carriage by age: a systematic review and meta-analysis. The Lancet Infectious diseases. 2010;10(12):853–61. Epub 2010/11/16. doi: 10.1016/S1473-3099(10)70251-6 . 21075057 10.1016/S1473-3099(10)70251-6

[9] Jeppesen CA, Snape MD, Robinson H, Gossger N, John TM, Voysey M, et al Meningococcal carriage in adolescents in the United Kingdom to inform timing of an adolescent vaccination strategy. The Journal of infection. 2015;71(1):43–52. Epub 2015/02/25. doi: 10.1016/j.jinf.2015.02.006 . 25709085 10.1016/j.jinf.2015.02.006

[10] de Greeff SC, de Melker HE, Spanjaard L, Schouls LM, van Derende A. Protection from routine vaccination at the age of 14 months with meningococcal serogroup C conjugate vaccine in the Netherlands. The Pediatric infectious disease journal. 2006;25(1):79–80. Epub 2006/01/06. . 16395110 10.1097/01.inf.0000195594.41449.c6

[11] de Voer RM, Mollema L, Schepp RM, de Greeff SC, van Gageldonk PG, de Melker HE, et al Immunity against Neisseria meningitidis serogroup C in the Dutch population before and after introduction of the meningococcal c conjugate vaccine. PloS one. 2010;5(8):e12144 doi: 10.1371/journal.pone.0012144 ; PubMed Central PMCID: PMC2921331. 20730091 10.1371/journal.pone.0012144 PMC2921331

[12] Ladhani SN, Beebeejaun K, Lucidarme J, Campbell H, Gray S, Kaczmarski E, et al Increase in Endemic Neisseria meningitidis Capsular Group W Sequence Type 11 Complex Associated With Severe Invasive Disease in England and Wales. Clinical infectious diseases: an official publication of the Infectious Diseases Society of America. 2015;60(4):578–85. doi: 10.1093/cid/ciu881 . 25389259 10.1093/cid/ciu881

[13] Lucidarme J, Scott KJ, Ure R, Smith A, Lindsay D, Stenmark B, et al An international invasive meningococcal disease outbreak due to a novel and rapidly expanding serogroup W strain, Scotland and Sweden, July to August 2015. Euro surveillance: bulletin Europeen sur les maladies transmissibles = European communicable disease bulletin. 2016;21(45). Epub 2016/12/06. doi: 10.2807/1560-7917.es.2016.21.45.30395 ; PubMed Central PMCID: PMCPMC5144941. 27918265 10.2807/1560-7917.ES.2016.21.45.30395 PMC5144941

[14] Campbell H, Saliba V, Borrow R, Ramsay M, Ladhani SN. Targeted vaccination of teenagers following continued rapid endemic expansion of a single meningococcal group W clone (sequence type 11 clonal complex), United Kingdom 2015. Euro surveillance: bulletin Europeen sur les maladies transmissibles = European communicable disease bulletin. 2015;20(28). Epub 2015/07/28. . 26212140 10.2807/1560-7917.es2015.20.28.21188

[15] Trotter CL, Borrow R, Findlow J, Holland A, Frankland S, Andrews NJ, et al Seroprevalence of antibodies against serogroup C meningococci in England in the postvaccination era. Clinical and vaccine immunology: CVI. 2008;15(11):1694–8. doi: 10.1128/CVI.00279-08 ; PubMed Central PMCID: PMC2583529. 18827191 10.1128/CVI.00279-08 PMC2583529

[16] Zhang Q, Finn A. Mucosal immunology of vaccines against pathogenic nasopharyngeal bacteria. Journal of clinical pathology. 2004;57(10):1015–21. Epub 2004/09/29. doi: 10.1136/jcp.2004.016253 ; PubMed Central PMCID: PMCPmc1770445. 15452151 10.1136/jcp.2004.016253 PMC1770445

[17] van Ravenhorst MB, van der Klis FRM, van Rooijen DM, Knol MJ, Stoof SP, Sanders EAM, et al Meningococcal serogroup C immunogenicity, antibody persistence and memory B-cells induced by the monovalent meningococcal serogroup C versus quadrivalent meningococcal serogroup ACWY conjugate booster vaccine: A randomized controlled trial. Vaccine. 2017 Epub 2017/07/03. doi: 10.1016/j.vaccine.2017.06.053 . 28668575 10.1016/j.vaccine.2017.06.053

[18] van Ravenhorst MB, van der Klis FRM, van Rooijen DM, Sanders EAM, Berbers GAM. Adolescent meningococcal serogroup A, W and Y immune responses following immunization with quadrivalent meningococcal A, C, W and Y conjugate vaccine: Optimal age for vaccination. Vaccine. 2017 Epub 2017/06/26. doi: 10.1016/j.vaccine.2017.06.007 . 28647167 10.1016/j.vaccine.2017.06.007

[19] Stoof SP, van der Klis FR, van Rooijen DM, Bogaert D, Trzcinski K, Sanders EA, et al Salivary antibody levels in adolescents in response to a meningococcal serogroup C conjugate booster vaccination nine years after priming: systemically induced local immunity and saliva as potential surveillance tool. Vaccine. 2015;33(32):3933–9. Epub 2015/06/24. doi: 10.1016/j.vaccine.2015.06.055 . 26100925 10.1016/j.vaccine.2015.06.055

[20] de Voer RM, van der Klis FR, Engels CW, Rijkers GT, Sanders EA, Berbers GA. Development of a fluorescent-bead-based multiplex immunoassay to determine immunoglobulin G subclass responses to Neisseria meningitidis serogroup A and C polysaccharides. Clinical and vaccine immunology: CVI. 2008;15(8):1188–93. doi: 10.1128/CVI.00478-07 ; PubMed Central PMCID: PMC2519294. 18550729 10.1128/CVI.00478-07 PMC2519294

[21] Lal G, Balmer P, Joseph H, Dawson M, Borrow R. Development and evaluation of a tetraplex flow cytometric assay for quantitation of serum antibodies to Neisseria meningitidis serogroups A, C, Y, and W-135. Clinical and diagnostic laboratory immunology. 2004;11(2):272–9. doi: 10.1128/CDLI.11.2.272-279.2004 ; PubMed Central PMCID: PMC371201. 15013975 10.1128/CDLI.11.2.272-279.2004 PMC371201

[22] Mitsi E, Roche AM, Reine J, Zangari T, Owugha JT, Pennington SH, et al Agglutination by anti-capsular polysaccharide antibody is associated with protection against experimental human pneumococcal carriage. Mucosal immunology. 2017;10(2):385–94. Epub 2016/09/01. doi: 10.1038/mi.2016.71 ; PubMed Central PMCID: PMCPMC5332540. 27579859 10.1038/mi.2016.71 PMC5332540

[23] Zhang Q, Lakshman R, Burkinshaw R, Choo S, Everard J, Akhtar S, et al Primary and booster mucosal immune responses to meningococcal group A and C conjugate and polysaccharide vaccines administered to university students in the United Kingdom. Infection and immunity. 2001;69(7):4337–41. Epub 2001/06/13. doi: 10.1128/IAI.69.7.4337-4341.2001 ; PubMed Central PMCID: PMCPmc98504. 11401971 10.1128/IAI.69.7.4337-4341.2001 PMC98504

[24] Zhang Q, Choo S, Everard J, Jennings R, Finn A. Mucosal immune responses to meningococcal group C conjugate and group A and C polysaccharide vaccines in adolescents. Infection and immunity. 2000;68(5):2692–7. Epub 2000/04/18. ; PubMed Central PMCID: PMCPmc97476. 10768961 10.1128/iai.68.5.2692-2697.2000 PMC97476

[25] Barnes GK, Workalemahu B, Kristiansen PA, Beyene D, Merdekios B, Fissiha P, et al Salivary and Serum Antibody Response Against Neisseria meningitidis After Vaccination With Conjugate Polysaccharide Vaccines in Ethiopian Volunteers. Scandinavian journal of immunology. 2016;84(2):118–29. Epub 2016/05/25. doi: 10.1111/sji.12451 . 27219622 10.1111/sji.12451

[26] Horton RE, Stuart J, Christensen H, Borrow R, Guthrie T, Davenport V, et al Influence of age and carriage status on salivary IgA to Neisseria meningitidis. Epidemiology and infection. 2005;133(5):883–9. doi: 10.1017/S0950268805004097 ; PubMed Central PMCID: PMC2870320. 16181509 10.1017/S0950268805004097 PMC2870320

[27] Marcotte H, Lavoie MC. Oral microbial ecology and the role of salivary immunoglobulin A. Microbiology and molecular biology reviews: MMBR. 1998;62(1):71–109. Epub 1998/04/08. ; PubMed Central PMCID: PMCPmc98907. 9529888 10.1128/mmbr.62.1.71-109.1998 PMC98907

[28] Zhang Z, Clarke TB, Weiser JN. Cellular effectors mediating Th17-dependent clearance of pneumococcal colonization in mice. The Journal of clinical investigation. 2009;119(7):1899–909. Epub 2009/06/11. doi: 10.1172/JCI36731 ; PubMed Central PMCID: PMCPMC2701860. 19509469 10.1172/JCI36731 PMC2701860

[29] Vann WF, Liu TY, Robbins JB. Bacillus pumilus polysaccharide cross-reactive with meningococcal group A polysaccharide. Infection and immunity. 1976;13(6):1654–62. Epub 1976/06/01. ; PubMed Central PMCID: PMCPMC420816. 184043 10.1128/iai.13.6.1654-1662.1976 PMC420816

[30] Filice GA, Hayes PS, Counts GW, Griffiss JM, Fraser DW. Risk of group A meningococcal disease: bacterial interference and cross-reactive bacteria among mucosal flora. Journal of clinical microbiology. 1985;22(2):152–6. Epub 1985/08/01. ; PubMed Central PMCID: PMCPMC268349. 3928679 10.1128/jcm.22.2.152-156.1985 PMC268349

[31] Brandtzaeg P. Secretory immunity with special reference to the oral cavity. Journal of oral microbiology. 2013;5 Epub 2013/03/15. doi: 10.3402/jom.v5i0.20401 ; PubMed Central PMCID: PMCPMC3595421. 23487566 10.3402/jom.v5i0.20401 PMC3595421

[32] Borrow R, Fox AJ, Cartwright K, Begg NT, Jones DM. Salivary antibodies following parenteral immunization of infants with a meningococcal serogroup A and C conjugated vaccine. Epidemiology and infection. 1999;123(2):201–8. ; PubMed Central PMCID: PMC2810750. 10579438 10.1017/s0950268899002915 PMC2810750

[33] Macpherson AJ, McCoy KD, Johansen FE, Brandtzaeg P. The immune geography of IgA induction and function. Mucosal immunology. 2008;1(1):11–22. Epub 2008/12/17. doi: 10.1038/mi.2007.6 . 19079156 10.1038/mi.2007.6

[34] Brandtzaeg P, Korsrud FR. Significance of different J chain profiles in human tissues: generation of IgA and IgM with binding site for secretory component is related to the J chain expressing capacity of the total local immunocyte population, including IgG and IgD producing cells, and depends on the clinical state of the tissue. Clinical and experimental immunology. 1984;58(3):709–18. Epub 1984/12/01. ; PubMed Central PMCID: PMCPMC1577102. 6439452 PMC1577102

[35] Dawes C. Physiological factors affecting salivary flow rate, oral sugar clearance, and the sensation of dry mouth in man. Journal of dental research. 1987;66 Spec No:648–53. Epub 1987/02/01. doi: 10.1177/00220345870660S107 . 3476629 10.1177/00220345870660S107

