# Supplementary material for: Induction of salivary antibody levels in Dutch adolescents after immunization with monovalent meningococcal serogroup C or quadrivalent meningococcal serogroup A, C, W and Y conjugate vaccine
Source: PLoS One. 2018 Apr 19;13(4):e0191261. doi: 10.1371/journal.pone.0191261 (PMC5908077; doi:10.1371/journal.pone.0191261)
Supplement: S1 Fig — Participants received a single vaccination with the Meningococcal serogroup C conjugated to tetanus toxoid (MenC-TT) vaccine or a quadrivalent Meningococcal serogroup A, C, W and Y conjugated to tetanus toxoid (MenACWY-TT) vaccine at enrolment. Blood abd saliva samples were collected before and 1 month and 1 year after this vaccination. ATP = According To Protocol. (DOC) [file pone.0191261.s001.doc]

**
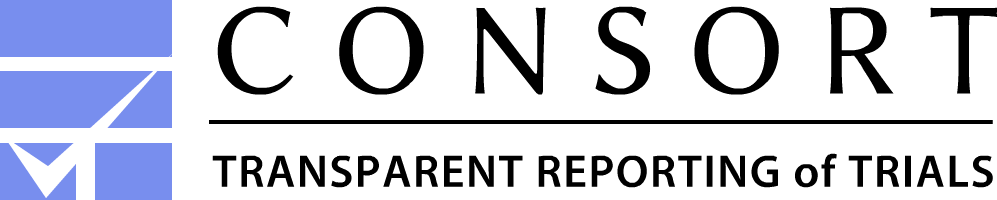
**

**CONSORT 2010 Flow Diagram**

**Allocation**

**+ 1 year**

**+1 month**

**Enrollment**

**Analysis**

Assessed for eligibility (n= 860)

Excluded (n= 344)

♦  Not meeting inclusion criteria (n=344)

♦  Other reasons (n=15)

Lost to follow-up (n=7)

No serum obtained for this time point (n=1)

Lost to follow-up (n=1)

No serum obtained for this time point (n=3)

Enrolled to receive the MenACWY-TT (n=246)

♦ Received allocated intervention (n=236)

♦ Did not receive allocated intervention because of fear for venapunction (n=10)

Lost to follow-up (n=5)

No serum obtained for this time point (n=1)

Enrolled to receive the MenC-TT (n=255)

♦ Received allocated intervention (n=253)

♦ Did not receive allocated intervention because of fear for venapunction (n=2)

Lost to follow-up (n=6)

No serum obtained for this time point (n=2)

)

Enrolled (n=501)

Included in the ATP (n=225)^[17, 18]^
Analysed for MenA (n=201), C (n=205), W (n=202) and Y (n=202)

Included in the ATP (n=239)^[17, 18]^
Analysed for MenC (n=216)
