# Supplementary material for: Induction of salivary antibody levels in Dutch adolescents after immunization with monovalent meningococcal serogroup C or quadrivalent meningococcal serogroup A, C, W and Y conjugate vaccine
Source: PLoS One. 2018 Apr 19;13(4):e0191261. doi: 10.1371/journal.pone.0191261 (PMC5908077; doi:10.1371/journal.pone.0191261)
Supplement: S1 Table — (DOCX) [file pone.0191261.s002.docx]

**S1 Table.** Correlation between serum and saliva serogroup-specific IgA and IgG concentrations per time point.

|  |  | **IgG** | | |  | **IgA** | | |
| --- | --- | --- | --- | --- | --- | --- | --- | --- |
| **Serogroup** | **Time point** | **Correlation coefficient** | **95% CI** | **P-value** |  | **Correlation coefficient** | **95% CI** | **P-value** |
| **MenA** | **T0** | 0.28 | 0.15-0.40 | <0.001 |  | 0.16 | 0.02-0.30 | 0.021 |
|  | **T1** | 0.53 | 0.42-0.62 | <0.001 |  | 0.34 | 0.21-0.45 | <0.001 |
|  | **T2** | 0.38 | 0.25-0.49 | <0.001 |  | 0.27 | 0.13-0.39 | <0.001 |
| **MenC** | **T0** | 0.43 | 0.35-0.51 | <0.001 |  | 0.34 | 0.26-0.43 | <0.001 |
|  | **T1** | 0.59 | 0.52-0.65 | <0.001 |  | 0.54 | 0.47-0.60 | <0.001 |
|  | **T2** | 0.63 | 0.56-0.68 | <0.001 |  | 0.36 | 0.28-0.44 | <0.001 |
| **MenW** | **T0** | 0.30 | 0.17-0.42 | <0.001 |  | 0.17 | 0.03-0.31 | 0.015 |
|  | **T1** | 0.70 | 0.62-0.76 | <0.001 |  | 0.34 | 0.21-0.46 | <0.001 |
|  | **T2** | 0.65 | 0.57-0.73 | <0.001 |  | 0.39 | 0.26-0.50 | <0.001 |
| **MenY** | **T0** | 0.41 | 0.28-0.51 | <0.001 |  | 0.06 | -0.08-0.20 | 0.404 |
|  | **T1** | 0.74 | 0.67-0.80 | <0.001 |  | 0.37 | 0.25-0.49 | <0.001 |
|  | **T2** | 0.77 | 0.70-0.82 | <0.001 |  | 0.43 | 0.31-0.53 | <0.001 |
