# Supplementary material for: Induction of salivary antibody levels in Dutch adolescents after immunization with monovalent meningococcal serogroup C or quadrivalent meningococcal serogroup A, C, W and Y conjugate vaccine
Source: PLoS One. 2018 Apr 19;13(4):e0191261. doi: 10.1371/journal.pone.0191261 (PMC5908077; doi:10.1371/journal.pone.0191261)
Supplement: S1 File — (PDF) [file pone.0191261.s003.pdf]

JIM-Study

Second meningococcal vaccination in Dutch children: Study to compare the tetravalent MenACWY-TT conjugate vaccine with the monovalent MenC-TT conjugate vaccine.

(In Dutch: "**J**uveniele **I**mmunisatie **M**eningokokken ACWY (**JIM**)-studie")

JIM-Study

PROTOCOL TITLE

Second meningococcal vaccination study in Dutch children: Study to compare the tetravalent MenACWY-TT conjugate vaccine with the monovalent MenC-TT conjugate vaccine.

Protocol ID	IMS-267
Short title	JIM-study
Version	2.1
Date	July, 2013
Coordinating investigator/project leader	1. Guy Berbers, PhD (CIb, RIVM)<br>RIVM<br>PO Box 1<br>3720 BA Bilthoven<br>Tel: +31 (0)30-274 2496<br>E-mail: Guy.Berbers@RIVM.nl<br>2. Fiona van der Klis, PhD<br>Tel: +31 (0)30-274 2272<br>E-mail: Fiona.van.der.Klis@RIVM.nl
Principal investigator(s) (in Dutch: hoofdonderzoeker/uitvoerder)	1. Mariette van Ravenhorst, PhD-student<br>Tel: +31 (0)6-20954479<br>E-mail: mariette.van.ravenhorst@gmail.com<br>2. Lieke Sanders, MD, PhD<br>Tel: +31 (0)30-274 2496<br>E-mail: L.Sanders@umcutrecht.nl
Sponsor (in Dutch: verrichter/opdrachtgever)	National Institute for Public Health and Environment (RIVM)<br>Antonie van Leeuwenhoeklaan 9<br>3721 MA Bilthoven<br>Postbus 1<br>3720 BA Bilthoven

Independent physician(s)	Tom Wolfs, MD, PhD<br>Tel: +31 (0)30-274 4980<br>E-mail: T.Wolfs@umcutrecht.nl
Laboratory sites	National Institute for Public Health and the Environment (RIVM)<br>Centre for Infectious Disease Control (CIb)<br>Laboratory for Infectious Diseases and Screening (LIS)<br>Antonie van Leeuwenhoeklaan 9<br>3721 MA Bilthoven

JIM-Study

PROTOCOL SIGNATURE SHEET

Name	Signature	Date
Sponsor or legal representative:<br>National Institute for Public Health and Environment (RIVM),<br>Centre for Infectious Disease Control, (CIb).<br><br>For non-commercial research,<br>Head of Department:<br>Roel Coutinho		
Coordinating Investigator/Project leader/<br>Dr. G.A.M. Berbers (CIb, RIVM)		
Principal Investigator 1:<br>Drs. M.B. van Ravenhorst (CIb, RIVM)		
Principal Investigator 2:<br>Dr. F.R. van der Klis (CIb, RIVM)		
Principal Investigator 3:<br>Prof. Dr. E.A.M. Sanders (WKZ, UMCU)		
Senior epidemiologist:<br>Mirjam Knol, PhD		

JIM-Study

1. INTRODUCTION AND RATIONALE 10
2. OBJECTIVES 16
3. STUDY DESIGN 18
4. STUDY POPULATION 20
    a. 4.1 Population (base) 20
    b. 4.2 Inclusion criteria 20
    c. 4.3 Exclusion criteria 20
    d. 4.4 Sample size calculation 21
5. TREATMENT OF SUBJECTS 23
    a. 5.1 Investigational product/treatment 23
    b. 5.2 Use of co-intervention 23
    c. 5.3 Escape medication 23
6. INVESTIGATIONAL MEDICINAL PRODUCTS 24
    a. 6.1 Name and description of investigational medicinal product 24
    b. 6.2 Summary of findings from non-clinical studies 24
    c. 6.3 Summary of findings from clinical studies 24
    d. 6.4 Summary of known and potential risks and benefits 24
    e. 6.5 Description and justification of route of administration and dosage 24
    f. 6.6 Dosages, dosage modifications and method of administration 24
    g. 6.7 Preparation and labeling of Investigational Medicinal Product 24
    h. 6.8 Drug accountability 25
7. METHODS 26
    a. 7.1 Study parameters/endpoints 26
        i. 7.1.1 Main study parameters 26
        ii. 7.1.2 Secondary study parameters 26
        iii. 7.1.3 Other study parameters 27
    b. 7.2 Randomization, blinding and treatment allocation 27
    c. 7.3 Study procedures 27
    d. 7.4 Withdrawal of individual subjects 28
    e. 7.5 Replacement of individual subjects after withdrawal 29
    f. 7.6 Follow-up of subjects withdrawn from treatment 29
    g. 7.7 Premature termination of the study 29
8. SAFETY REPORTING 30
    a. 8.1 Section 10 WMO event 30
    b. 8.2 Adverse and serious adverse events 30
    c. 8.3 Follow-up of adverse events 31
    d. 8.4 Data Safety Monitoring Board (DSMB) 31
9. STATISTICAL ANALYSIS 32
10. ENTHICAL CONSIDERATIONS 35
    a. 10.1 Regulation statement 35

b. 10.2 Recruitment and informed consent 35
c. 10.3 Objection by minors 35
d. 10.4 Benefits and risks assessment 36
e. 10.5 Compensation for injury 36
f. 10.6 Incentives 36
11. ADMINISTRATIVE ASPECTS AND PUBLICATION 37
a. 11.1 Handling and storage of data and documents 37
b. 11.2 Amendments 37
c. 11.3 Annual progress report 38
d. 11.4 End of study report 38
e. 11.5 Public disclosure and publication policy 38
12. REFERENCES 39

LIST OF ABBREVIATIONS AND RELEVANT DEFINITIONS

**ABR**	ABR form, General Assessment and Registration form, is the application form that is required for submission to the accredited Ethics Committee (In Dutch, ABR = Algemene Beoordeling en Registratie)
**AE**	Adverse Event
**AR**	Adverse Reaction
**CA**	Competent Authority
**CCMO**	Central Committee on Research Involving Human Subjects; in Dutch: Centrale Commissie Mensgebonden Onderzoek
**DSMB**	Data Safety Monitoring Board
**EU**	European Union
**EudraCT**	European drug regulatory affairs Clinical Trials
**GCP**	Good Clinical Practice
**IB**	Investigator's Brochure
**IC**	Informed Consent
**IMP**	Investigational Medicinal Product
**IMPD**	Investigational Medicinal Product Dossier
**MenY**	Meningococcal serogroup Y
**MenC-TT**	Meningococcal serogroup A tetanus toxoid conjugate vaccine
**MenACWY-TT**	Meningococcal serogroup A, C, W and Y tetanus toxoid conjugate vaccine
**MenC-PS**	Meningococcal serogroup C polysaccharide vaccine
**METC**	Medical research ethics committee (MREC); in Dutch: medisch ethische toetsing commissie (METC)
**MIA**	Fluorescent-bead-based multiplex immunoassay
**NIP**	National Immunization Program (in Dutch: Rijks Vaccinatie Programma, RVP)
**RIVM**	National Institute for Public Health and the Environment (in Dutch: Rijksinstituut voor Volksgezondheid en Milieu
**(S)AE**	(Serious) Adverse Event
**SBA**	Serum Bactericidal Antibody Assay
**SPC**	Summary of Product Characteristics (in Dutch: officiële productinfomatie IB1-tekst)
**Sponsor**	The sponsor is the party that commissions the organization or performance of the research, for example a pharmaceutical company, academic hospital, scientific organization or investigator. A party that provides funding for a study but does not commission it is not regarded as the sponsor, but referred to as a subsidizing party.
**SUSAR**	Suspected Unexpected Serious Adverse Reaction
**TT**	Tetanus toxoid
**Wbp**	Personal Data Protection Act (in Dutch: Wet Bescherming Persoonsgevens)
**WMO**	Medical Research Involving Human Subjects Act (in Dutch: Wet Medisch-wetenschappelijk Onderzoek met Mensen)

JIM-Study

SUMMARY

Rationale: Neisseria meningitides is a gram-negative diplococcal bacterium that causes septicemia and meningitis. Meningococcal disease occurs comprehensively world-wide. The different serogroups are geographically distributed around the world.

The incidence of meningococcal serogroup Y (MenY) appears to increase throughout countries in Europe, including the Netherlands. In the Netherlands, MenY accounted for 12% of invasive meningococcal disease cases in 2012, an increase from 2% in 2006. Therefore, when the incidence of invasive MenY disease in Europe continues to increase, it can lead to serious public health problems.

Nowadays, many young adults go travelling world-wide. A tetravalent MenACWY tetanus toxoid conjugate vaccine (MenACWY-TT) is available in the Netherlands, offering protection against serogroups A, C, W and Y. In the Netherlands, some of the young travelers get already the tetravalent MenACWY-TT vaccine.

An increase in the relative proportion and the absolute numbers of Meningococcal serogroup C (MenC) invasive meningococcal disease during the 1990s led to vast media attention, increasing public anxiety and the governmental decision to include a MenC-TT vaccination into the Dutch National Immunization Program (NIP). In addition to the implementation of a MenC-TT vaccination in the Dutch NIP in September 2002, a catch-up campaign was conducted between June and November 2002 during which all children between 1 and 18 years were invited to receive a single MenC-TT vaccination. In the past years it has become clear that protection induced by a primary MenC-TT vaccination appears to be age-dependent. It is clear that the current single vaccination at 14 months is not sufficient to maintain long lasting protection against MenC and that there is a need for a second vaccination at an older age.

Even though the increase of MenC in 1999/2000 was much more notable, the MenACWY-TT vaccine may be beneficial for a second vaccination at older age in the future. This second vaccination will protect the adolescents and maintain the herd immunity that persists up until today.

Currently, a MenACWY-TT vaccination in adolescence is considered in many countries. However, longitudinal effectiveness studies with the MenACWY-TT vaccine for a second meningococcal vaccination are lacking. Therefore, the evaluation of persistence of antibodies, after a booster vaccination with MenACWY-TT is critical to monitor the duration of protection of a MenACWY-TT conjugate vaccine in adolescence after priming with MenC-TT at age of 14 months.

Objective: The aim of this study is to investigate the immune response to the tetravalent MenACWY-TT vaccine administered as a second meningococcal vaccination and compare the booster response to MenC with the booster response to the monovalent MenC-TT conjugate vaccine. This study will generate important data for the decision which vaccine can be used for a second meningococcal vaccination in the Dutch NIP, and for the determination of the appropriate age for a possible tetravalent MenACWY-TT conjugate vaccination after priming with MenC-TT at young age.

Study design: Intervention study.

Study population: Participants eligible to this study are healthy Dutch children who received all regular vaccinations according to the NIP. Five groups will be included; n=80 per group. All children must have received a primary MenC-TT vaccination at an earlier age, either at the age of 14 months (regular vaccination time point as part of the NIP; 10- and 12-year olds), or during the mass catch-up campaign in 2002 (15-years olds).

Intervention: Participants will receive the vaccination with either the registered tetravalent vaccine (Nimenrix; 0,5mL) or the registered monovalent vaccine (NeisVac-C™; 0,5 mL) at the first visit. Blood and saliva samples will be drawn prior to the vaccination (T0), 1 month (T1) and 1 year (T2) after the vaccination.

Main study parameters: Functional antibody levels against MenA, MenC, MenW and MenY are measured using the Serum Bactericidal Antibody assay (SBA). In addition, serum and salivary MenA-PS, MenC-PS, MenW-PS and MenY-PS specific IgG and IgA levels and serum IgG subclasses and avidity are measured using fluorescent-bead-based multiplex immunoassay (MIA).

Nature and extent of the burden and risks associated with participation, benefit and group relatedness: Participants benefit from participating in the study by receiving an additional MenACWY-TT or MenC-TT vaccination. From the public health perspective, participation in this study will contribute to the improvement of the National Immunization Programme (NIP). Vaccination and venapunctures might be painful and unpleasant. On request of the participant, Xylocainespray can be used to reduce possible local pain during the venapunction. Nimenrix and NeisVac-C™ are registered vaccines in the Netherlands. Mild adverse reactions to one of the vaccine components may occur but they are expected to be mainly local and transient. Severe allergic reactions to one of the vaccine components are unlikely to occur. As a compensation for the vaccination and the venapunctures, all participants will receive a total of €25, - in vouchers.

1. INTRODUCTION AND RATIONALE

Neisseria meningitides is a gram-negative diplococcal bacterium that causes septicemia and meningitis.[1] Neisseria meningitides is carried in the nasopharynx and transmission occurs mainly through very close contact via respiratory droplets and saliva. The overall carriage in infants is low (4-5%), then subsequently increases to a peak of 24% in 19-year olds and then decreases in adulthood to approximately 7% in 50-year olds.[2]

As the bacterium is a commensal, meningococcal infection can be asymptomatic and disappears normally after several days to months. However, acquisition of Neisseria meningitides can lead to local inflammation, invasion of mucosal surfaces, access to the bloodstream and development of rapidly progressive meningitis and/or sepsis.[1] Meningococcal disease can be life threatening, and although Neisseria meningitides is susceptible for antibiotics, morbidity (such as hearing loss, scarring and amputation of limbs) and mortality rates of invasive disease remain high. The overall mortality rate in meningococcal disease is still between 10 and 15%.[1,3] Therefore, meningococcal disease remains an important public health problem.

Neisseria meningitides is a strictly human pathogen. Based on the capsular groups, 13 different serotypes of Neisseria meningitides have been identified, but only six (A, B, C, W, X and Y) are associated with invasive disease.[1]

Meningococcal disease occurs comprehensively world-wide. The different serogroups are geographically distributed around the world. (Figure 1)[4,5]

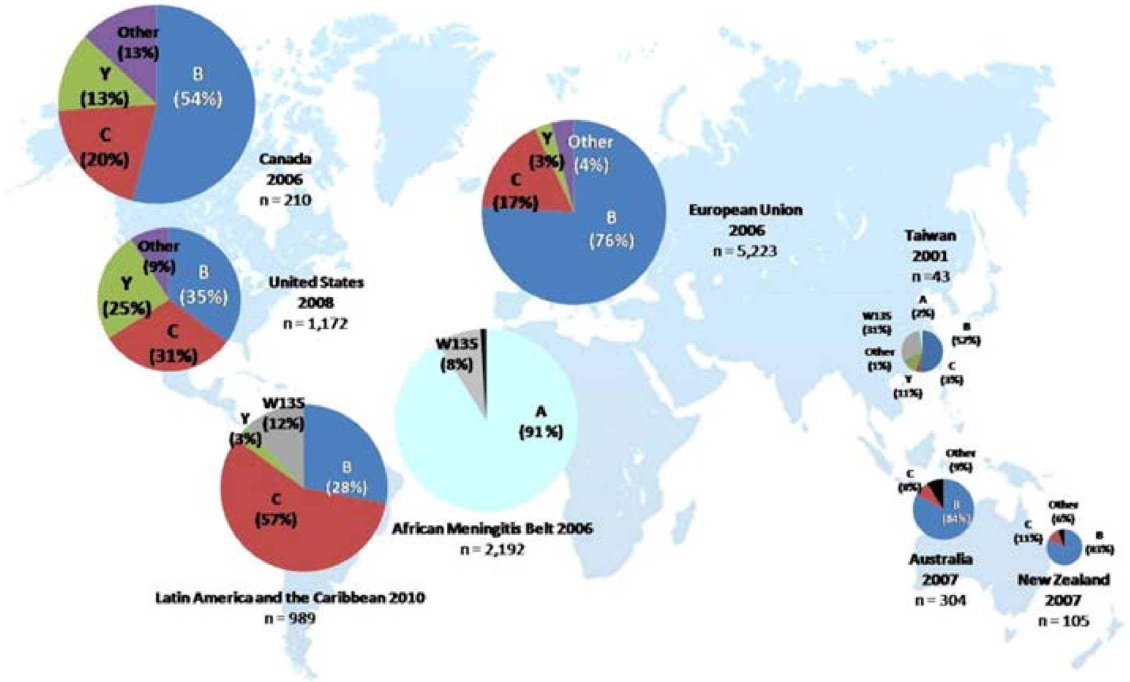

Figure 1. Proportion of meningococcal disease by serogroup by geographic region.[4]

In sub-Saharan Africa, the so-called “meningitis-belt”, recurrent epidemics of serogroup A occur. Serogroups B and C are the most prevalent in the US and Europe. Serogroup W was not considered of great epidemiologic importance until an outbreak at the Hajj pilgrimage. Serogroup Y has caused since the mid 1990s increased rates of disease in the US.[6]

In the Netherlands Meneningococcal serogroup B (MenB) is now responsible for 76% of meningococcal disease. Currently, a vaccine against MenB is not yet available in the Netherlands. Recent epidemiological surveillance indicated an increase of the proportion of serogroup Y (MenY) of invasive meningococcal disease in some parts of Europe (see Figure 2).[6] In the Netherlands, MenY accounted for 12% of invasive meningococcal disease cases in 2012, an increase from 2% in 2006.

Country	N	%
Estonia	0	0%
Greece	0	0%
Iceland	0	0%
Ireland	0	0%
Malta	0	0%
Portugal	0	0%
Slovakia	0	0%
Slovenia	0	0%
Spain	2	1%
Poland	5	2%
Austria	4	5%
France	26	5.5%
Czech Republic	4	6%
Denmark	1	6%
Germany	22	7%
United Kingdom	68	7%
Netherlands	11	8%
Italy	11	10%
Switzerland	20	21%
Norway	13	31%
Finland	13	38%
Sweden	23	39%

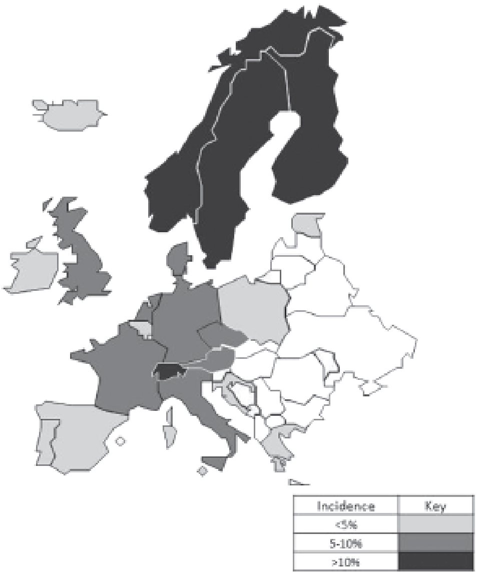

Figure 2. Relative proportion of Neisseria meningitides serogroup Y in various European countries in 2010. (Broker et al. 2012)

Although MenY has disproportionately affected older adults, in France, the average age of MenY patients declined from 76 y in 2005 to 21 y in 2010.[1] This shift coincided with an increase in overall MenY cases in Europe.[6] The age distribution of MenY disease in the Netherlands is shown in Figure 3.

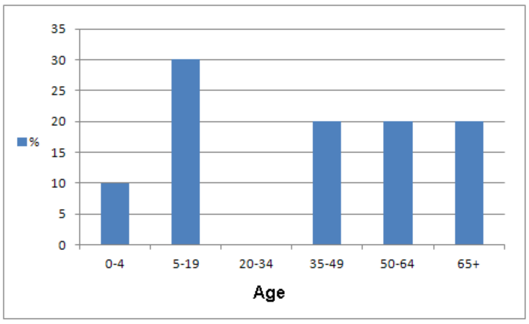

Figure 3. Age distribution of MenY disease in the Netherlands, 2012

Nowadays, many young adults go travelling world-wide. A tetravalent MenACWY tetanus toxoid conjugate vaccine (MenACWY-TT, Nimenrix) is available in the Netherlands, offering protection against serogroups A, C, W and Y. In the Netherlands, some of the young travelers get already the MenACWY-TT vaccine.

In 1995/1996 the incidence of Meningococcal serogroup C (MenC) disease in the Netherlands was approximately 0.35 per 100.000 inhabitants and was constant over the years. However, by the end of the 90's the incidence of MenC disease suddenly increased throughout Europe. In 2000/2001 the incidence of MenC disease in the Netherlands had increased to 1.17 per 100.000 inhabitants. This led to vast media attention, increasing public anxiety and the governmental decision to include a MenC vaccination into the Dutch National Immunization Program (NIP).

In addition to the implementation of a MenC-polysaccharide conjugated to tetanus toxoid vaccination (MenC-TT) in the Dutch NIP in September 2002, a catch-up campaign was conducted between June and November 2002 during which all children between 1 and 18 years were invited to receive a single MenC-TT vaccination. Overall vaccine coverage was 94% in the mass campaign and afterwards MenC disease disappeared in the vaccinated cohorts and even decreased dramatically in the non-immunized cohorts.[7]

After vaccine introduction, the incidence of MenC disease decreased substantially in both immunised and non-immunized individuals.[8,9] It is suggested that this great success of the MenC-TT vaccine is primarily based on the catch-up campaign inducing large scale herd immunity by reducing the nasopharyngeal carriage of MenC bacteria in the population. The present low incidence of MenC disease is considered to be a consequence of the induced herd-immunity and to a much lesser degree to individual immunity.[10] Sustained immunization of a large part of the population should therefore be pursued.

Unfortunately, it became clear recently that vaccine-induced immunity after primary immunization is not sustained in young children.[11-14] Meningococcal serogroup C polysaccharide (MenC-PS) specific IgG antibody concentrations decline rapidly after vaccination in children < 5 years of age. This is associated with a reduction in the proportion of children with a serum bactericidal antibody (SBA) level above the accepted correlate of protection of ≥ 8. (Figure 4)[11,13,15]

In contrast to the children vaccinated during infancy, children vaccinated at older ages appear to have a better and longer-lasting antibody response. (figure 4A) This coincides with a higher percentage of children with SBA levels above the correlate of protection. (figure 4B)[11]

It is unknown how long the herd immunity for MenC disease will last. However, it is clear that the current single vaccination at 14 months is not sufficient to maintain long lasting protection against MenC and that there is a need for a second vaccination at an older age. Without a second MenC-TT vaccination, children vaccinated at 14 months will reach the second period of increased risk for invasive MenC disease (12-18 years) with low serologic levels of protective immunity. Recently a study has started in the Netherlands to establish which age would be most appropriate to implement a second MenC-TT vaccination (Second Immunization MenC (Dutch acronym: "TIM"-study). Preliminary results show a good immune response to a booster MenC-TT vaccination. The Joint Committee on Vaccination an Immunisation in England (JCVI) has suggested that immunization with a booster dose of one meningococcal C conjugate vaccine in adolescence, following a single dose in childhood (given as part of the 1999/2000 catch up campaign in England) produces a long-lasting elevation of SBA titres.[16]

A.

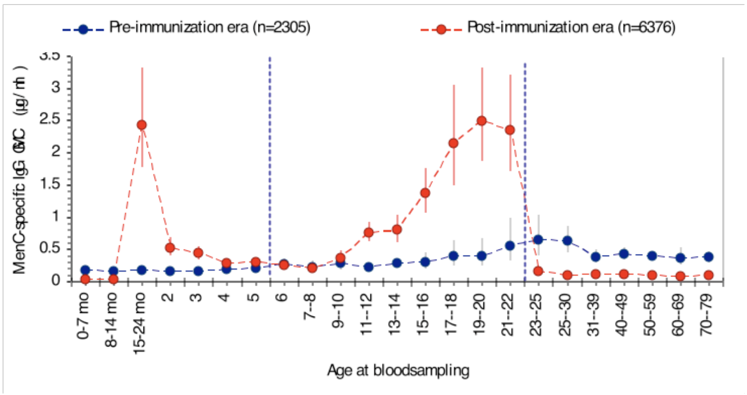

B.

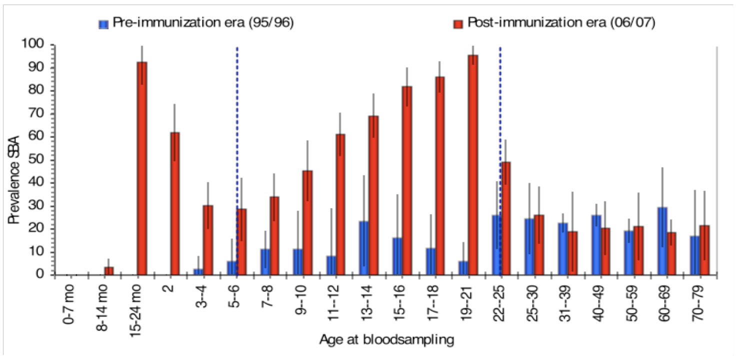

Figure 4. MenC-PS specific IgG and serum bactericidal antibody levels in Dutch population. MenC PS-specific IgG (A) and seroprevalence of SBA titers ≥8 (B) within each age-cohort, pre- (1995/1996, in blue) and 5 years post (2006/2007, in red) introduction of the MenC conjugate vaccine in 2002. Error bars indicate 95% confidence intervals. Area between vertical lines indicates cohorts that were immunized in catch-up campaign in 2002. Age at blood sampling is stated in years unless indicated otherwise (mo = age in months)

Currently, a MenACWY vaccination in adolescence is considered in many countries, because of the observed increase of meningococcal disease cases due to MenY. Even though the increase of MenC in 1999/2000 was much more notable, the tetravalent MenACWY-TT vaccine may be beneficial for a second vaccination at older age in the NIP to prevent upcoming MenY disease in the future. The Advisory Committee on Immunization Practices (ACIP) in the US recommends routine administration of a single MenACWY vaccine for all persons aged 11 or 12 years, with a booster dose at age of 16 years. However, in the US routine vaccination against meningococcal disease is not recommended for children at young ages.[17] The JCVI advised that the cost-effectiveness of a MenACWY conjugate vaccination in place of second MenC-TT vaccination in adolescence should be undertaken.[16] Longitudinal effectiveness studies with the MenACWY-TT vaccine for a second meningococcal vaccination are lacking. Therefore, the evaluation of persistence of antibodies, after a booster vaccination with MenACWY-TT is critical to monitore the duration of protection of a MenACWY-TT conjugate vaccine in adolescence after priming with MenC-TT at age of 14 months.

In conclusion, with an increasing incidence of MenY and increasing number of travelers among the young population, the use of the tetravalent MenACWY-TT vaccine may be beneficial for the second meningococcal vaccination at an older age in the future. This second vaccination will protect the adolescents and maintain the herd immunity that persists up until today.

The aim of this study is to investigate the immune response to the tetravalent MenACWY-TT vaccine administered as a second meningococcal vaccination and compare the booster response to MenC with the booster response to the monovalent MenC-TT conjugate vaccine. We want to establish whether MenC antibody responses after MenACWY-TT vaccination in adolescence primed with the monovalent MenC-TT vaccine are non-inferior to responses after a second vaccination with the monovalent MenC-TT vaccine. The TIM-study was designed to determine an appropriate age for a second MenC-TT vaccination in the Dutch NIP. With the current study we want to determine whether the tetravalent MenACWY-TT vaccine can also be used for second vaccination at an older age. Furthermore, we would like to establish whether there is a similar age difference in the antibody response to MenA, W and Y after vaccination, as was previously found for MenC (Figure 4).

This study will generate important data for the decision which vaccine can be used for a second meningococcal vaccination in the Dutch NIP, and for the determination of the appropriate age for a possible tetravalent MenACWY-TT conjugate vaccination after priming with MenC-TT at young age.

For this study, we chose a similar design as used for the TIM-study to compare the study results (head to head comparison) and include age groups of 10-, 12- and 15- year olds. In the TIM-study, the 10 year olds were primed at the age of 14 months, and the 12- and 15 year olds were primed during the catch-up campaign in 2002. In this study, both the 10- and 12- years olds will be primed at the age of 14 months. The 15-years olds will be primed during the mass campaign in 2002 at the age of 2 or 3 (depending on the start of this study). It is expected that the age at priming can be of influence for the antibody responses. Therefore, we need two groups - one for vaccination with the monovalent MenC-TT vaccine and one for vaccination with the tetravalent MenACWY-TT vaccine - for each age category in this study. As in both studies the 10 year olds were primed at the age of 14 month we will not include a group of 10-year olds for vaccination with the monovalent MenC-TT vaccine, but use the results from this age group from the TIM-study instead. The comparison of the age groups between the JIM-study and the TIM-study are presented in figure 5.

"TIM"-study

Age	Age of priming	Booster
10 years	14 months	MenC-TT
12 years	1 years (mass campaign)	MenC-TT
15 years	4 years (mass campaign)	MenC-TT

"JIM"-study

Age	Age of priming	Booster
10 years	14 months	MenACYW-TT
10 years	14 months	MenC-TT
12 years	14 months	MenACYW-TT
12 years	14 months	MenC-TT
15 years	2 years (mass campaign)	MenACYW-TT
15 years	2 years (mass campaign)	MenC-TT

Figure 5. Study groups of the “TIM”-study and the “JIM”-study.

The MenC-TT vaccine has been demonstrated to be more immunogenic than vaccines with the MenC-polysaccharide conjugated to diphtheria toxoid or diphtheria mutated toxoid CRM-197.[15,18] This vaccine is currently used in the Dutch NIP and was used in the TIM-study. Therefore, it is expected that the MenACWY-TT vaccine is also more immunogenic than the other tetravalent meningococcal vaccines that are currently available.[19-20] So, we will use this tetravalent MenACWY-TT vaccine for the JIM-study.

2. **OBJECTIVES**

The aim of this study is to investigate the immune response to a tetravalent MenACWY-TT vaccine in 10-, 12- and 15-year old children primed with the monovalent MenC-TT conjugate vaccine at a young age and to

1. determine whether the MenC-specific antibody response after vaccination with a tetravalent MenACWY-TT vaccine is not inferior to MenC specific antibody responses after vaccination with the monovalent MenC-TT conjugate vaccine.
2. determine whether there is an age-dependent difference in the primary response to MenA, MenW and MenY after vaccination with the tetravalent vaccine, as was previously found for MenC.
3. determine the appropriate age for a tetravalent MenACWY-TT conjugate vaccination after priming with MenC-TT at young age.

2.1 Primary objectives

The primary objective is to demonstrate non-inferiority of SBA levels against MenC at 1 year (T2) after vaccination in the group vaccinated with tetravalent MenACWY-TT vaccine as compared with the group vaccinated with monovalent MenC-TT conjugate vaccine in 10-, 12-, and 15-years old children.

If non-inferiority is demonstrated, the objective is to compare SBA levels against MenA, MenW and MenY at 1 year (T2) after vaccination between the three age groups that are vaccinated with tetravalent MenACWY-TT vaccine.

2.2 Secondary objectives

- To compare SBA levels against MenC at 1 month (T1) between the vaccine groups within the three age groups.
- To compare SBA levels against MenC of ≥8 (persistence of vaccine induced protective antibody levels) at 1 month (T1) and 1 year (T2) between the vaccine groups within the three age groups.
- To compare serum MenC-PS specific IgG levels at 1 month (T1) and 1 year (T2) between the vaccine groups within the three age groups.
- To compare the decay rate of SBA levels and MenC-PS specific IgG levels after secondary vaccination (i.e. the difference between T2 and T1) between the vaccine groups within the three age groups.
- To compare SBA levels against MenA, MenW and MenY at 1 month (T1) between the three age groups within the MenACWY-TT vaccine group.
- To compare SBA levels against MenA, MenW and MenY of ≥8 at 1 month (T1) and 1 year (T2) between the three age groups within the MenACWY-TT vaccine group.
- To compare serum MenA-PS, MenY-PS and MenW-PS specific IgG levels at 1 month (T1) and 1 year (T2) between the three age groups within the MenACWY-TT vaccine group.
- To compare serum IgG antibody levels against tetanus, the carrier protein for both vaccines, at 1 month (T1) and 1 year (T2)? between the vaccine groups within the three age groups.

- To compare serum IgA levels against MenA, MenC, MenW and MenY at 1 month (T1) and at 1 year (T2) between the vaccine groups within the three age groups.
- To compare MenC-PS specific IgG subclasses (IgG1/IgG2 ratio) and avidity at 1 month (T1) and 1 year (T2)? between the vaccine groups within the three age groups.
- To compare SBA and IgG levels against MenC at 1 month and 1 year between the MenC-TT group of the current study and the TIM-study for the the 12- and 15- year olds, to establish the effect of the age at priming on antibody responses to a second MenC-TT vaccination during adolescence.
- Explorative: To measure saliva IgG and IgA levels at T0 1 month (T1) and 1 year (T2) in all groups.
- Explorative: To measure B- and T-cell memory immune responses at T0, 1 month (T1) and 1 year (T2) in all groups.

3. STUDY DESIGN

This is an intervention study to investigate the immune response to the tetravalent MenACWY-TT vaccine administered as a second meningococcal vaccination and compare the booster response to MenC with the booster response with the monovalent MenC-TT conjugate vaccine. The effect of the tetravalent MenACWY-TT and a second MenC-TT will be investigated in three age-groups: 10-, 12- and 15 year olds.

3.1 Recruitment

The participants will be recruited from Utrecht (Leidsche Rijn) and the surrounding region of Utrecht (e.g. Nieuwegein, Zeist, Houten, Ijsselstein, Bilthoven and Vleuten). Addresses from eligible children (based on their age at the start of the study) will be attained through the 'Regionale Coordinatie Programma's (RCP).' An invitation letter will be sent to the parents of all potential participants (Annex 1: Invitation Letter). This invitation letter includes brief information about the study and a reply card with the question whether or not the parents and child are willing to participate in the study and want more information. Based on former similar studies conducted by the RIVM, a participation percentage of 5-10% can be expected. We would like to include 80 participants per group (see section 4.4 for sample size calculation). The invitation letter will therefore be sent to ca. 6500 potential participants in the above mentioned region.

After receiving the reply card that indicates that a child and its parent(s) are willing to participate, the principal investigator will contact the (parents of the) child to give more information and to check whether the child is eligible for inclusion in the study based on the inclusion and exclusion criteria (see 4.2 and 4.3). Afterwards an extensive information letter (Annex 2: Patient Information Letter) together with an informed consent form (Annex 3: Informed Consent) will be sent to the potential participant. Within one week after sending the information, the principal investigator will contact the (parents of the) child a second time to answer additional questions. If parents and child remain willing to participate, an appointment is made for the first visit at a study site close to where the potential participant lives. During this first visit, the informed consent form will be signed by the principal investigator. The participant and his/her parents are asked to sign the informed consent form in advance to ensure that both parents signed the form. Afterwards, the study will start.

3.1.1 Study sites

In every city or village where participants are recruited, a local study site will be set up (e.g. local public health centers). This will ensure that the participants do not have to travel too far to visit the study site.

3.2 Vaccination and collection of blood and saliva samples

The total duration of the study is one year and comprises three visits from the participants to a study site. All participants will receive the tetravalent MenACWY-TT vaccination (they have been primed at an earlier age) or their second MenC-TT vaccination at the first visit. Blood samples will be drawn prior to this vaccination (T0) and 1 month (28-42 days, T1) and 1 year (+/- 2 weeks, T2) after the vaccination. In addition, saliva samples will be obtained at T0, T1 and T2. See Table 1 for an overview of the study schedule. The study will start as soon as possible and end one year later.

Table 1. Study calendar

Visit number at study site	Actions
T0: First visit	• Sign informed consent form<br>• Draw first blood sample<br>• Draw first saliva sample<br>• Administer MenACWY-TT vaccination or second MenC-TT vaccination
T1: Second visit (1 month after T0)	• Draw second blood sample<br>• Draw second saliva sample
T2: Final visit (1 year after T0)	• Draw final blood sample<br>• Draw final saliva sample

From most participants, one tube of 5 mL blood per visit will be drawn. This blood sample will be used to attain quantitative and qualitative information on the antibody response that is evoked by the MenACWY-TT vaccination or the second MenC-TT vaccination. In order to study cellular responses after MenACWY-TT vaccination or MenC-TT, an additional 16 mL of blood is needed. All of the participants will be asked (on the informed consent form) for permission for drawing this additional 16 mL (2 tubes of 8 mL) of blood at all visits. This comes down to a total of 3 tubes per visit instead of one. To collect these 2 additional tubes, there is <u>no</u> extra venapuncture needed. The aim is to take these additional tubes at 20 children per group.

4. STUDY POPULATION

4.1 Population (base)

Five groups will be recruited:

- Group 1: 10 years olds (male + female, n=80, with tetravalent MenACWY-TT vaccine)
- Group 2: 12 year olds (male + female, n=80, with tetravalent MenACWY-TT vaccine)
- Group 3: 12 year olds (male + female, n=80, with monovalent MenC-TT vaccine)
- Group 4: 15 year olds (male + female, n=80, with tetravalent MenACWY-TT vaccine)
- Group 5: 15 year olds (male + female, n=80, with monovalent MenC-TT vaccine)

The participants will be recruited from Utrecht (Leidsche Rijn) and the surrounding region of Utrecht (e.g. Nieuwegein, Zeist, Houten, Ijsselstein, Bilthoven and Vleuten).

4.2 Inclusion criteria

Participants are 10-, 12, and 15-year old children who have received a primary vaccination with a single dose of MenC-TT vaccine (NeisVac-C™) either during the mass catch-up campaign in 2002 (group 4 and 5) or at the age of 14 months (regular vaccination time point since 2002 according to the Dutch NIP; group 1,2 and 3). Furthermore, participants have to fulfil all of the following criteria:

- Provision of written informed consent by both parents and (if child is 12 or 15 years old; see Annex 3) child (including giving permission to inform the GP);
- Good general health;
- Received all regular vaccines according to Dutch NIP;
- Adherent to protocol, and available during the study period

4.3 Exclusion criteria

Any of the following criteria at the start of the study will exclude a volunteering child from participation:

- Severe acute (infectious) illness or fever (>38.5°C) within 14 days before vaccination;
- Antibiotic use within 14 days of enrollment;
- Present evidence of serious disease(s) demanding (immunosuppressive) medical treatment that might interfere the results of the study within the last 3 months (like corticosteroids, chronic infection, bleeding disorder, immune dysfunction, genetic anomaly);
- Known or suspected allergy to any of the vaccine components (by medical history);
- Occurrence of serious adverse event after primary MenC-TT vaccination or other vaccination (by medical history)
- Known or suspected immune deficiency;
- History of any neurologic disorder, including epilepsy;
- Previous administration of plasma products (including immunoglobulins) within the last 6 months;
- Pregnancy;
- Previous confirmed or suspected meningococcal disease;
- Former received doses of MenC vaccines in addition to the primary vaccination;
- Former received any tetravalent MenACWY vaccination;
- Received any vaccination in the past month.

Presence of the in- and exclusion criteria (including pregnancy) will be checked by interviewing the parent about the medical history of the child during the first telephone call. In addition, presence of the exclusion criteria (including pregnancy) will be checked by interviewing the parent and child at the first study visit, prior to signing the informed consent form.

4.4 Sample size calculation

Non-inferiority between vaccine groups – GMT ratio

The objective is to demonstrate non-inferiority of SBA levels against MenC at 1 year after vaccination in the group vaccinated with tetravalent MenACWY-TT vaccine as compared with the group vaccinated with monovalent MenC-TT conjugate vaccine in 10-, 12-, and 15-years old children.

For the sample size calculation we use the following measures:

- NI margin of 0.67 (1 divided by 1.5), meaning that the lower limit of the 95% confidence interval of the ratio of the SBA GMT in the MenACWY-TT group and the MenC-TT group should not exceed 0.67.
- Standard deviation of ln(SBA level) of 0.92 (based on results of the TIM study where the same SBA MenC assay was used that will be used in the current study) .
- Significance level (alpha) of 0.05 one-sided.
- Power of 0.80.

The sample size calculation shows that we need 64 children per vaccine group in each age group.

Superiority between age groups – GMT ratio

The objective is to study differences in SBA levels against MenA, MenW and MenY at 1 year after vaccination between the three age groups that are vaccinated with tetravalent MenACWY-TT vaccine.

For the sample size calculation we use the following measures:

- A difference in GMT ratio of 2.0 between the age groups.
- Standard deviation of ln(SBA level) against MenA, MenW and MenY of 0.87, 1.05 and 1.26 (based on Borja-Tabora et al[19], Table 2), for the calculation we take 1.26 because this is the highest value.
- Significance level (alpha) of 0.05/3 two-sided (Bonferroni correction for 3 comparisons).
- Power of 0.80.

The sample size calculation shows that we need 69 children in each age group.

Table 2. rSBA one year after tetravalent MenACWY-TT vaccination in age group 11-17 years.[19]

Antibody	N	rSBA GMT	Lower limit	Upper limit	SE (ln titer)	Sd (ln titer)
A	218	2369.1	2111.5	2658.0	0.058	0.866964
C	218	1966.5	1660.4	2329.0	0.086	1.274522
Y	218	4943.7	4298.3	5686.1	0.071	1.053896

**W**	219	3676.7	3111.5	4344.6	0.085	1.260259

Sample size

Assuming that 10% of the participants will leave the study, and blood sampling will fail in +/- 2 participants per group, we aim to include 80 participants per group.

5. TREATMENT OF SUBJECTS

5.1 Investigational product/treatment

During this study the subjects will receive one dose of a tetravalent MenACWY-TT or monovalent MenC-TT. Tetravalent MenACWY-TT (registration number: EU/1/12/767/001/-007): Nimenrix. One dose of 0.5 mL for intramuscular injection contains: 5 microgram of Neisseria meningitidis group A polysaccharide, 5 microgram of Neisseria meningitidis group C polysaccharide, 5 microgram of Neisseria meningitidis group W polysaccharide and 5 microgram of Neisseria meningitidis group Y polysaccharide. The polysaccharides are conjugated to 44 microgram of tetanus toxoid. Other additive products are sodium chloride and water for injections.

Monovalent MenC-TT (registration number RVG 26343): NeisVac-C™. One dose of 0.5 mL for intramuscular injection contains 10 microgram of Neisseria meningitidis group C (strain C11) polysaccharide (de-O-acetylated). The polysaccharide is conjugated to 10-20 micrograms of tetanus toxoid and is absorbed to aluminium hydroxide (0.5 mg). Other additive products are sodium chloride and water for injections.

5.2 Use of co-intervention

Not applicable

5.3 Escape medication

Not applicable

6. INVESTIGATIONAL MEDICINAL PRODUCT

6.1 Name and description of investigational medicinal product

During this study the subjects will receive one dose of a tetravalent MenACWY-TT or monovalent MenC-TT. Tetravalent MenACWY-TT (registration number: EU/1/12/767/001/-007): Nimenrix. One dose of 0.5 mL for intramuscular injection contains: 5 microgram of Neisseria meningitidis group A polysaccharide, 5 microgram of Neisseria meningitidis group C polysaccharide, 5 microgram of Neisseria meningitidis group W polysaccharide and 5 microgram of Neisseria meningitidis group Y polysaccharide. The polysaccharides are conjugated to 44 microgram or tetanus toxoid. Other additive products are sodium chloride and water for injections.

Monovalent MenC-TT (Registration number RVG 26343): NeisVac-C™. One dose of 0.5 mL for intramuscular injection contains 10 microgram of Neisseria meningitidis group C (strain C11) polysaccharide (de-O-acetylated). The polysaccharide is conjugated to 10-20 micrograms of tetanus toxoid and is absorbed to aluminium hydroxide (0.5 mg). Other additive products are sodium chloride and water for injections.

The vaccines will only be used in the study if released by the manufacturer and the appropriate authorities.

6.2 Summary of findings from non-clinical studies

See Investigators Brochures (Annex 4A+4B) Nimenrix and NeisVac-C™.

6.3 Summary of findings from clinical studies

See Investigators Brochures (Annex 4A+4B) Nimenrix and NeisVac-C™.

6.4 Summary of known and potential risks and benefits

See patient information leaflets (Annex 5) and Investigators Brochures (Annex 4A+4B) of Nimenrix and NeisVac™.

6.5 Description and justification of route of administration and dosage

The Nimenrix vaccine (0.5 mL) or NeisVac-C™ vaccine (0.5 mL) is injected intramuscularly in the upper arm. This is a customary and well-accepted route of administration of this vaccine. See Investigators Brochures (Annex 4A and 4B).

6.6 Dosages, dosage modifications and method of administration

All study subjects will receive one intramuscular injection with one dosage of the vaccine Nimenrix or NeisVac-C™.

6.7 Preparation and labelling of Investigational Medicinal Product

See Investigators Brochures (Annex 4A+4B.), and the SOP for vaccination (Annex 6)

6.8 Drug accountability

Vaccines will be provided by GSK (tetravalent Nimenrix, about 250 vaccines) and Baxter (monovalent NeisVac-C™, about 170 vaccines). Vaccines are stored and transported at 2-8˚C (see page 8 of Investigators Brochures (Annex 4A+4B). The number of given vaccinations will be listed on the study location.

7. METHODS

7.1 Study parameters/endpoints

Blood and saliva samples will be collected from all participants at three time points: prior to the vaccination (T0) and 1 month (T1) and 1 year afterwards (T2). Saliva supernatants, serum and PBMCs will be isolated from these samples in order to determine the following parameters:

7.1.1 Main study parameters

7.1.1.1 Serum Bactericidal Antibody assay (SBA)

SBA levels are a measure for MenC, MenA, MenW and MenY functional antibody activity. An SBA level of ≥ 8 is considered as a good correlate of protection against invasive meningococcal disease and this threshold was extended to the other serogroups as well.[19,21] SBA levels are expressed as the reciprocal of the final serum dilution yielding ≥ 50% killing at 60 minutes.[11] In addition, the geometric mean titers (GMT) of SBAs will be determined and used for comparison between groups (see section 9.1 and 9.2).

7.1.1.2 Serum MenACWY-PS specific IgG antibodies, -subclasses and –avidity levels

In order to achieve the primary and secondary objectives, geometric mean concentrations, the fluorescent-bead-based multiplex immunoassay (MIA) will be used to measure subclasses and avidity of MenA, C, W and Y polysaccharide (PS) specific IgG antibodies.

7.1.2 Secondary study parameters

7.1.2.1 Serum and salivary Meningococcal specific IgA

IgA is the major antibody at mucosal surfaces and considered to be important in limiting meningococcal colonisation and preventing early invasion. Serum and salivary MenA-PS, MenC-PS, MenW-Ps and MenY-PS specific IgA levels can be measured in order to investigate their correlation and the (longitudinal) kinetics of local and systemic IgA production after primary MenACWY-TT and MenC-TT vaccination and secondary MenC-vaccination.

7.1.2.2 Meningococcal specific B-cell and T-cell responses

Part of the participants will be asked for an additional 16 mL of blood for the purpose of studying cellular responses (see section 3.2). This blood will be collected in vacutainer cell preparation tubes (CPT) and used for the isolation of PBMCs.[22] PBMCs will be divided in purified B- cell populations and T- cell populations. B-cells will be cultured and memory B-cells will be polyclonally stimulated.[23] After stimulation, B- cell memory responses will be measured against MenC-PS. T-cell cultures will be stimulated with tetanus toxoid (TT), TT-MenC conjugate without alum or MenC-TT. After stimulation, detection of IFN-γ secreting T-cells will be performed.[24,25] Explorative: B- and T-cell memory immune responses will be measured against MenA-PS, MenW-PS and MenY-PS.

7.1.3 Other study parameters

During the course of the study it could be possible that additional parameters turn out be of interest, such as antibody levels other than the ones mentioned here, or certain cytokine levels. These parameters will then be measured using the most suitable laboratory tests available at the RIVM. If newly developed and better laboratory tests become available, these will be used wherever possible.

7.2 Randomisation, blinding and treatment allocation

Group 2, 3, 4 and 5 will be classified by randomization by computer using a random number generator (www.random.org). Just before the vaccination is given, the computer will be selected which vaccine is given by the study team member. The study team member should be vaccinate the subject with the product as indicated by computer. . The treatment product should be recorded in the case report form (CRF Annex 9) by the study team member.

Field Code Changed

7.3 Study procedures

7.3.1 Invasive study procedures

The invasive study procedures (vaccination and venapunctures) will be carried out by experienced and qualified persons according to standard operating procedures. A local anesthetic (Xylocainespray) can be used to minimize the pain of the venapuncture.

7.3.1.1 Vaccination

See standard operating procedure (SOP) of vaccination (Annex 6)

7.3.1.2 Venapuncture

See SOP of venapuncture (Annex 7)

7.3.1.3 Saliva sampling

See SOP of saliva sampling (Annex 8)

7.3.2 Laboratory tests

7.3.2.1 Serum Bactericidal Antibody (SBA) assay

The target strains use in the SBA assays are L10 for serogroup A, C11 for serogroup C, M01 240070 for serogroup W-135, and M03 241125 for serogroup Y.[19,26] SBA titers are expressed as the reciprocal of the final serum dilution yielding ≥ 50% killing at 60 minutes[11] and as geometric mean titers (GMT).

7.3.2.3 Avidity assay

To assess avidity of specific IgG antibodies, serum samples will be incubated with ammonium thiocyanate (NH_4SCN, 0.5M), in order to dissociate low-avidity antigen-antibody binding, or with PBS. See reference de Voer et al. 2009 for the procedure.[22] The level of avidity of IgG antibodies will be expressed as the avidity index (AI). This is the

percentage of IgG antibodies that remains bound to MenA, MenC, MenW or MenY PS-conjugated beads after treatment with NH_4SCN (measured with MIA) and is calculated as follows:

$$AI = \frac{\text{(IgG concentration after incubation with } NH_4SCN)}{\text{(IgG concentration after incubation with PBS)}} \times 100\%$$

An AI of 0-33% is arbitrarily indicated as low, 33-66% as intermediate and 66-100% as high.[24,27]

7.3.2.2 Fluorescent-bead-based mult()iplex immunoassay (MIA)

The fluorescent-bead-based multiplex immunoassay (MIA) will be used to measure anti-MenA-PS, anti-MenC-PS, anti-MenW-PS and anti-MenY-PS specific IgG antibody concentrations using CDC1992 reference serum as standard. See reference de Voer RM et al, 2008 for the detailed procedure.[28] The MIA will also be used to measure MenA-PS, MenC-PS, MenW-PS and MenY-PS specific IgG subclasses and avidity[28] to measure serum and salivary IgA and to measure IgG antibody levels against tetanus, the carrier protein for the MenA, MenC, MenW and MenY polysaccharide in the conjugate vaccines.[29] Finally, the MIA will also be used to measure cytokine levels from the B- and T-cell supernatants.

7.3.2.4 ELIspot

B-cell memory responses against MenA-PS, MenC-PS, MenW-PS and MenY-PS will be measured by ELIspot assays. See reference de Voer et al. 2009 for the procedure.[22] T-cell cultures will be stimulated with tetanus toxoid (TT), TT-MenA, TT-MenC, TT-MenW or TT-MenY conjugate without alum or MenA-, MenC-, MenW- or MenY-PS. After stimulation, detection of IFN-γ secreting T-cells will also be done through ELIspot assays.[24,25]

7.4 Withdrawal of individual subjects

Subjects can leave the study at any time for any reason if they wish to do so without any consequences. The investigator can decide to withdraw a subject from the study for urgent medical reasons.

7.5 Replacement of individual subjects after withdrawal

Not applicable.

7.6 Follow-up of subjects withdrawn from treatment

Not applicable.

7.7 Premature termination of the study

Nimenrix is a registered vaccine which has already been used in the Netherlands (and other countries). NeisVac-C™ is also a registered vaccine in the Netherlands and has already been used in the Netherlands (and other countries) in the same age group (children 10, 12 and 15 years of age). It is therefore unlikely that serious side effects will occur that can lead to premature termination of the study.

8. SAFETY REPORTING

8.1 Section 10 WMO event

In accordance to section 10, subsection 1, of the WMO, the investigator will inform the participants and the reviewing accredited METC if anything occurs, on the basis of which it appears that the disadvantages of participation may be significantly greater than was foreseen in the research proposal. The study will be suspended pending further review by the accredited METC, except insofar as suspension would jeopardise the participants' health. The investigator will take care that all participants are kept informed.

8.2 Adverse and serious adverse events

Adverse events (AEs) are defined as any undesirable experience occurring to a participant during the study, whether or not considered related to the vaccine, the vaccination or the venapuncture. All adverse events reported spontaneously by the participant, his/her parent(s) or observed by the principal investigator or her staff will be recorded in the CRF (Annex 9).

A serious adverse event (SAE) is any untoward medical occurrence or effect that at any dose of the vaccine:

- results in death;
- is life threatening (at the time of the event);
- requires hospitalisation or prolongation of existing inpatients' hospitalisation;
- results in persistent or significant disability or incapacity;
- is a new event of the trial likely to affect the safety of the participant, such as an unexpected outcome of an adverse reaction, major safety finding from a newly completed animal study, etc.

SAEs will lead to definite suspension of the study participant.

All SAEs will be reported by the principal investigator through the web portal ToetsingOnline to the accredited METC that approved the protocol, within 15 days after the sponsor has first knowledge of the serious adverse reactions. SAEs that result in death or are life threatening should be reported expedited. The expedited reporting will occur not later than 7 days after the principal investigator has first knowledge of the adverse reaction. This is for a preliminary report with another 8 days for completion of the report.

All SAEs with a suspected (probable or definite) relationship to the vaccine (as indicated by the responsible investigator) will be reported to Lareb and the Medicines Evaluation Board (CBG) by the sponsor.

8.2.1 Suspected unexpected serious adverse reactions (SUSAR)

Adverse reactions are all untoward and unintended responses to an investigational product related to any dose administered. Unexpected adverse reactions are adverse reactions, of which the nature, or severity, is not consistent with the applicable product information (e.g. Investigator's Brochure for an unapproved IMP or Summary of Product Characteristics (SPC) for an authorised medicinal product).

The sponsor will report expedited the following SUSARs through the web portal ToetsingOnline to the METC:

- SUSARs that have arisen in the clinical trial that was assessed by the METC;
- SUSARs that have arisen in other clinical trials of the same sponsor and with the same medicinal product, and that could have consequences for the safety of the subjects involved in the clinical trial that was assessed by the METC.

The remaining SUSARs are recorded in an overview list (line-listing) that will be submitted once every half year to the METC. This line-listing provides an overview of all SUSARs from the study medicine, accompanied by a brief report highlighting the main points of concern. The expedited reporting of SUSARs through the web portal ToetsingOnline is sufficient as notification to the competent authority.

The sponsor will report expedited all SUSARs to the competent authorities in other Member States, according to the requirements of the Member States.

The expedited reporting will occur not later than 15 days after the sponsor has first knowledge of the adverse reactions. For fatal or life threatening cases the term will be maximal 7 days for a preliminary report with another 8 days for completion of the report.

All SUSARs will also be reported to the CBG and the distributor of the vaccine.

8.2.2 Annual safety report

In addition to the expedited reporting of SUSARs, the sponsor will submit, once a year throughout the clinical trial, a safety report to the accredited METC and competent authority.
This safety report consists of:

– a list of all suspected (unexpected or expected) serious adverse reactions, along with an aggregated summary table of all reported serious adverse reactions, ordered by organ system, per study;
– a report concerning the safety of the subjects, consisting of a complete safety analysis and an evaluation of the balance between the efficacy and the harmfulness of the medicine under investigation.

8.3 Follow-up of adverse events

All adverse events will be followed until they have abated, or until a stable situation has been reached. Depending on the event, follow up may require additional tests or medical procedures as indicated, and/or referral to the general physician or a medical specialist.

8.4 Data Safety Monitoring Board (DSMB)

Not applicable.

9. STATISTICAL ANALYSIS

Data analysis will be performed by the principal and sponsor investigators. Validated data sets will be used for analysis.

9.1 Intention-to-treat (ITT) and per-protocol (PP) population

The ITT population will include all children. The PP population will include children who had their blood samples taken at 28-42 days after vaccination and at 1 year (+- 14 days) after vaccination. All analyses will be performed on the ITT and PP population.

9.2 Baseline characteristics

Baseline characteristics including sex, age at first and second vaccination, age at blood sampling, serum and saliva SBA, IgG and IgA levels before adolescence vaccination will be presented for the two vaccine groups and the three age groups. Categorical variables will be presented as percentages and continuous variables will be presented as means with standard deviation or, if not normally distributed, as medians with minimum and maximum. Within the vaccine groups, the age groups will be compared with regard to the baseline characteristics. Differences in categorical variables will be tested with a chi-square test. Differences in continuous variables will be tested with a t-test if normally distributed, and with a Mann-Whitney test if not normally distributed.

9.3 Primary analysis

Geometric mean titers (GMTs) with 95% confidence intervals of the SBA levels against MenC will be calculated for the five study groups at the 1 year time point. The difference in the GMTs (GMT ratio) with one-sided 95% confidence interval will be calculated between the group vaccinated with tetravalent MenACWY-TT vaccine and the group vaccinated with monovalent MenC-TT vaccine for the 10- (MenC-TT group from TIM study), 12- and 15-year olds. Non-inferiority will be concluded if the lower limit of the 95% confidence interval is not smaller than 0.67 (i.e. the NI margin). A p-value for non-inferiority will be calculated.

Geometric mean titers (GMTs) with 95% confidence intervals of the SBA levels against MenA, MenW and MenY will be calculated for the three age groups vaccinated with the tetravalent MenACWY-TT vaccine at the 1 year time point. Differences in GMTs between the age groups will be tested with ANOVA and corrected for 3 comparisons. If there are differences in baseline characteristics between the age groups which could influence the GMTs, we will adjust for these variables by means of linear regression.

9.4 Secondary analyses

- Geometric mean titers (GMTs) with 95% confidence intervals of the SBA levels against MenC will be calculated for the five study groups at the 1 month time point. Differences in GMTs between the vaccine groups will be tested with a t-test for each age group (for the 10-year olds the MenC-TT group from the TIM study will be used for comparison).
- Percentage of children with SBA level ≥8 against MenC with 95% confidence interval will be calculated for the five study groups at 1 month and 1 year. Differences in percentage of protection

between the vaccine groups will be tested with a chi-square test or, in case of low numbers in certain cells, with a Fisher's Exact test for each age group (for the 10-year olds the MenC-TT group from the TIM study will be used for comparison).

- Geometric mean concentrations (GMCs) with 95% confidence intervals of the IgG levels against MenC will be calculated for the five study groups at 1 month and 1 year. Differences in GMCs between the vaccine groups will be tested with a t-test for each age group (for the 10-year olds the MenC-TT group from the TIM study will be used for comparison).
- The decline in SBA and IgG levels against MenC between 1 year and 1 month after vaccination will be calculated for the five study groups. Differences in this decline between the vaccine groups will be tested with a t-test for each age group (for the 10-year olds the MenC-TT group from the TIM study will be used for comparison).
- Geometric mean titers (GMTs) with 95% confidence intervals of the SBA levels against MenA, MenW and MenY will be calculated for the three age groups vaccinated with the tetravalent MenACWY-TT vaccine at the 1 month time point. Differences in GMTs between the age groups will be tested with ANOVA and corrected for 3 comparisons. If there are differences in baseline characteristics between the age groups which could influence the GMTs, we will adjust for these variables by means of linear regression.
- Percentage of children with SBA level ≥8 against MenA, MenW and MenY with 95% confidence interval will be calculated for the three age groups vaccinated with the tetravalent MenACWY-TT vaccine at 1 month and 1 year. Differences in percentage of protection will be tested with a chi-square test or, in case of low numbers in certain cells, with a Fisher's Exact test, and corrected for 3 comparisons. If there are differences in baseline characteristics between the age groups which could influence the percentages, we will adjust for these variables by means of log-binomial regression.
- Geometric mean concentrations (GMCs) with 95% confidence intervals of the IgG levels against MenA, MenW and MenY will be calculated for the three age groups vaccinated with the tetravalent MenACWY-TT vaccine at 1 month and 1 year. Differences in GMCs between the age groups will be tested with ANOVA and corrected for 3 comparisons. If there are differences in baseline characteristics between the age groups which could influence the GMCs, we will adjust for these variables by means of linear regression.
- Geometric mean concentrations (GMCs) with 95% confidence intervals of the IgG levels against tetanus will be calculated for the five study groups at 1 month and 1 year. Differences in GMCs between the vaccine groups will be tested with a t-test for each age group (for the 10-year olds the MenC-TT group from the TIM study will be used for comparison).
- Geometric mean concentrations (GMCs) with 95% confidence intervals of the IgA levels against MenA, MenC, MenW and MenY will be calculated for the five study groups at 1 month and 1 year. Differences in GMCs between the vaccine groups will be tested with a t-test for each age group (for the 10-year olds the MenC-TT group from the TIM study will be used for comparison).
- Geometric mean concentrations (GMCs) with 95% confidence intervals of the IgG subclasses levels against MenC will be calculated for the five study groups at 1 month and 1 year. Differences in GMCs

between the vaccine groups will be tested with a t-test for each age group (for the 10-year olds the MenC-TT group from the TIM study will be used for comparison).

  - Mean avidity indices and standard deviation for MenC will be calculated for the five study groups at 1 month and 1 year. Differences in avidity indices between the vaccine groups will be tested with a t-test for each age group (for the 10-year olds the MenC-TT group from the TIM study will be used for comparison).

- Differences in GMT/GMCs of SBA and IgG levels against MenC between the groups vaccinated with MenC-TT vaccine in the current study and in the TIM-study will be tested with a t-test for for the 12- and 15- year olds.

10. ETHICAL CONSIDERATIONS

10.1 Regulation statement

This clinical study will be performed according to the current rules for Good Clinical Practice (GCP), as described by the Committee for Proprietary Medical Products (CPMP) of the European Union and the International Committee on Harmonization (ICH) in "Note for Guidance on Good Clinical Practice, document CPMP/ICH/135/95", effective since January 17th 1997 and according to the Dutch Medical Research Involving Human Subjects Act (WMO), under the general ruling of the Clinical Trial Directive of the EU (2001/20/EU). These rules include the ethical guidelines described in the "Declaration of Helsinki" (World Medical Association Declaration of Helsinki: 'Ethical Principles for Medical Research involving Human Subjects'. Adopted by the 18th World Medical Association (WMA), Helsinki, Finland, 1964; amended by the 29th WMA, Tokyo, Japan, 1975; 35th WMA, Venice, Italy, 1983; 41st WMA, Hong Kong, 1989; the 48th WMA, Sommerset West, Republic of South Africa, 1996; 52nd WMA, Edinburgh, Scotland, 2000; 53rd WMA Washington, USA, 2002; 55th WMA Tokyo, Japan, 2004 and the 59th WMA General Assembly in Seoul, 2008).

10.2 Recruitment and informed consent

The participants will be recruited from Utrecht (Leidsche Rijn) and the surrounding region of Utrecht (e.g. Nieuwegein, Zeist, Houten, Ijsselstein, Bilthoven and Vleuten). Addresses from eligible children (based on their age at the start of the study) will be attained through the 'Regionale Coordinatie Programma's (RCP).' An invitation letter will be sent to the parents of all potential participants (Annex 1: Invitation Letter). This invitation letter includes brief information about the study and a reply card with the question whether or not the parents and child are willing to participate in the study and want more information. Based on former similar studies conducted by the RIVM, a participation percentage of 5-10% can be expected. The invitation letter will therefore be sent to +/-6500 potential participants in the above mentioned region.

After receiving the reply card that indicates that a child and its parent(s) are willing to participate, the principal investigator will contact the (parents of the) child to give more information and to check whether the child is eligible for inclusion in the study based on the inclusion and exclusion criteria. Afterwards an extensive information letter (Annex 2: Patient Information Letter) together with an informed consent form (Annex 3: Informed Consent) will be sent to the potential participant. Within one week after sending the information, the principal investigator will contact the (parents of the) child a second time to answer additional questions. If parents and child are willing to participate, an appointment is made for the first visit at a study site close to where the potential participant lives. During this first visit, the informed consent form will be signed by the principal investigator. The participant and his/her parents are asked to sign the informed consent form in advance to ensure that <u>both</u> parents signed the form. Afterwards, the study will start.

10.3 Objection by minors

All participants are minors. If during the course of the study one of the participants objects to voluntary participation to (one of) the study procedures (e.g. vaccination of venapuncture), the code 'gedragscode verzet minderjarigen' (WMO, Article. 4, lid 1) will be followed.

10.4 Benefits and risks assessment

Participants benefit from participating in the study by receiving an additional MenACWY-TT or MenC-TT vaccination which theoretically provides increased protection against MenA, MenC, MenY and/or MenW invasive disease. This second MenACWY-TT or MenC-TT vaccination is currently not routinely administered to these age-groups according to the NIP. From the public health perspective, participation in this study will contribute to the improvement of the National Immunization Programme (NIP).

Vaccination and venapunctures might be painful and unpleasant. Nonetheless, they are relatively low risk invasive procedures. On request of the participant, Xylocainespray can be used to reduce possible local pain during the venapuncture.

Nimenrix and NeisVac-C™ are registered vaccines in the Netherlands. Mild adverse reactions to the vaccine may occur but they are expected to be mainly local and transient (see Investigators Brochure Annex 4A+4B). NeisVac-C™ has already been used in the Netherlands (and other countries) in the same age group (children 10, 12 and 15 years of age) during the catch-up campaign in 2002. During the catch-up campaign over 3 million children were vaccinated and only 1512 developed and adverse reaction. All adverse reactions are described in the RIVM rapport 240082001/2004 ‘Ervaringen met bijwerkingen van de eenmalige Meningokokken C-vaccinatiecampagne in 2002.’ (Annex 4C) Of the 1512 adverse reactions reported, 41 were considered serious. All these 41 children recovered completely and the mass vaccination campaign with NeisVac-C™ was described as extremely safe. Thereby, Nimenrix is used for a travelers vaccination in the Netherland and is safely tested by children (0-18 years of age).[19,20]

We also consider it very unlikely that a more adverse reaction will occur after a second meningococcal vaccination, because this is not seen in other countries (e.g. UK, Spain, Greece, Ireland, Iceland and Portugal) which have a 2+1 vaccination scheduled for MenC vaccination. Thereby, in the TIM study the MenC-TT vaccination was used for a second meningococcal vaccination without the occurrence of more adverse reactions.

Severe allergic reactions to one of the vaccine components are unlikely to occur; the chance of such an event to occur will reasonably not be larger than found after injection of other vaccines. The vaccine will only be used in the study if released by the manufacturer and the appropriate authorities.

As a compensation for the vaccination, the venapunctures and the saliva sampling, all participants will receive a total of €25, - in vouchers after completion of the study (see section 10.6).

10.5 Compensation for injury

According to a Ministerial Order, RIVM is excluded from compulsory insurance for clinical research as determined by the Dutch law on Medical Investigations (WMO, section 7, paragraph 6). Participants can recover the loss from RIVM. Any claims will be settled according to the terms of an insurance company. Participants will be informed about these terms in detail in the Patient Information Letter (see Annex 2).

10.6 Incentives

Participants will receive a voucher of €10, - after the second blood sampling and an additional voucher of €15, - after the last blood sampling.

11. ADMINISTRATIVE ASPECTS AND PUBLICATION

11.1 Handling and storage of data and documents

All study data will be registered in a collection of files (the source documents) and handled confidentially. Separate records will be made for each participant. Parents and child can declare whether they wish to receive a report on the level of protection the child developed after the second MenACWY-TT or MenC-TT vaccination. These reports will be provided by the principal investigator. The principal investigator assures that the anonymity of the participants is maintained; keeping separate files of codes, names and addresses of participants. All clinical data on participants obtained from the handling, treatment (vaccination and venapuncture) and observation will be recorded on Case Report Forms (CRF Annex 9). The CRF forms the basis for further analysis of the study results. Personal identifiers will not be recorded on the CRF, with exception of date of birth, initials and gender. In addition, each participant is assigned a unique code (UTN number). To enable efficient data analysis, electronic data files will be created. Source documents and hard copies of electronic files/analyses will be stored according to GCP guidelines (for a period of 15 years if permission is obtained in the informed consent form).

11.1.1 Case Report Form

The CRF will contain information obtained according to the study assessments described in the previous chapters. The sponsor investigator is not entitled to know personal data of the participant. Thus, the principal clinical investigator is required to separate personal and study data on the CRF. Throughout the CRF, on every page, the UTN number will be used as the unique participant identifier. Furthermore, date and time of study procedures and assessments will be recorded throughout the CRF for all recorded observations (see Annex 9).

11.1.2 Data Entry Procedures

Data registered in the source documents will be made available for analysis in electronic data files. To establish a validated data set for analysis, a procedure of entry and verification will be used. All necessary changes and corrections after data entry will be motivated, dated and signed by the investigators.

11.2 Amendments

A ‘substantial amendment’ is defined as an amendment to the terms of the METC application, or to the protocol or any other supporting documentation, that is likely to affect to a significant degree:

- the safety or physical or mental integrity of the subjects of the trial;
- the scientific value of the trial;
- the conduct or management of the trial; or
- the quality or safety of any intervention used in the trial.

All substantial amendments will be notified to the accredited METC and to the competent authority.
Non-substantial amendments will not be notified to the accredited METC and the competent authority, but will be recorded and filed by the sponsor.

11.3 Annual progress report

The investigator/sponsor will submit a summary of the progress of the trial to the accredited METC once a year. Information will be provided on the date of inclusion of the first subject, numbers of subjects included and numbers of subjects that have completed the trial, serious adverse events/ serious adverse reactions, other problems, and amendments.

11.4 End of study report

The principal investigator/sponsor will notify the accredited METC of the end of the study within a period of 90 days. The end of the study is defined as the last patient's last visit. In case the study is ended prematurely, the investigator will notify the accredited METC, including the reasons for the premature termination.
Within one year after the end of the study, the principal investigator/sponsor will submit a final study report with the results of the study, including any publications/abstracts of the study, to the accredited METC.

11.5 Public disclosure and publication policy

The study results will be reported in an internal report and submitted for publication in peer-reviewed journals. Publications will be drafted by the sponsor investigators.

12. REFERENCES

1. STEPHENS, D.S., GREENWOOD, B. and BRANDTZAEG, P., 2007. Epidemic meningitis, meningococcaemia, and Neisseria meningitidis. *Lancet,* 369(9580), pp. 2196-2210.

2. CHRISTENSEN, H., MAY, M., BOWEN, L., HICKMAN, M. and TROTTER, C.L., 2010. Meningococcal carriage by age: a systematic review and meta-analysis. *The Lancet infectious diseases,* 10(12), pp. 853-861.

3. ROUPHAEL, N.G. and STEPHENS, D.S., 2012. Neisseria meningitidis: biology, microbiology, and epidemiology. *Methods in molecular biology (Clifton, N.J.),* 799, pp. 1-20.

4. HALPERIN, S.A., BETTINGER, J.A., GREENWOOD, B., HARRISON, L.H., JELFS, J., LADHANI, S.N., MCINTYRE, P., RAMSAY, M.E. and SAFADI, M.A., 2012. The changing and dynamic epidemiology of meningococcal disease. *Vaccine,* 30 Suppl 2, pp. B26-36.

5. HARRISON, L.H., TROTTER, C.L. and RAMSAY, M.E., 2009. Global epidemiology of meningococcal disease. *Vaccine,* 27 Suppl 2, pp. B51-63.

6. BROKER, M., JACOBSSON, S., DETORA, L., PACE, D. and TAHA, M.K., 2012. Increase of meningococcal serogroup Y cases in Europe: a reason for concern? *Human vaccines & immunotherapeutics,* 8(5), pp. 685-688.

7. VAN DER ENDE A, SPANJAARD L., 2010. Bacterial meningitis in the Netherlands; Annual Report 2009. *Amsterdam: Netherlands Reference Laboratory for Bacterial Meningitis (AMC/RIVM),* .

8. CAMPBELL, H., ANDREWS, N., BORROW, R., TROTTER, C. and MILLER, E., 2010. Updated postlicensure surveillance of the meningococcal C conjugate vaccine in England and Wales: effectiveness, validation of serological correlates of protection, and modeling predictions of the duration of herd immunity. *Clinical and vaccine immunology : CVI,* 17(5), pp. 840-847.

9. CAMPBELL, H., BORROW, R., SALISBURY, D. and MILLER, E., 2009. Meningococcal C conjugate vaccine: the experience in England and Wales. *Vaccine,* 27 Suppl 2, pp. B20-9.

10. MAIDEN, M.C., IBARZ-PAVON, A.B., URWIN, R., GRAY, S.J., ANDREWS, N.J., CLARKE, S.C., WALKER, A.M., EVANS, M.R., KROLL, J.S., NEAL, K.R., ALA'ALDEEN, D.A., CROOK, D.W., CANN, K., HARRISON, S., CUNNINGHAM, R., BAXTER, D., KACZMARSKI, E., MACLENNAN, J., CAMERON, J.C. and STUART, J.M., 2008. Impact of meningococcal serogroup C conjugate vaccines on carriage and herd immunity. *The Journal of infectious diseases,* 197(5), pp. 737-743.

11. DE VOER, R.M., MOLLEMA, L., SCHEPP, R.M., DE GREEFF, S.C., VAN GAGELDONK, P.G., DE MELKER, H.E., SANDERS, E.A., BERBERS, G.A. and VAN DER KLIS, F.R., 2010. Immunity against Neisseria meningitidis serogroup C in the Dutch population before and after introduction of the meningococcal c conjugate vaccine. *PloS one,* 5(8), pp. e12144.

12. ISHOLA, D.A.,JR, BORROW, R., FINDLOW, H., FINDLOW, J., TROTTER, C. and RAMSAY, M.E., 2012. Prevalence of serum bactericidal antibody to serogroup C Neisseria meningitidis in England a decade after vaccine introduction. *Clinical and vaccine immunology : CVI,* 19(8), pp. 1126-1130.

13. SNAPE, M.D., KELLY, D.F., GREEN, B., MOXON, E.R., BORROW, R. and POLLARD, A.J., 2005. Lack of serum bactericidal activity in preschool children two years after a single dose of serogroup C meningococcal polysaccharide-protein conjugate vaccine. *The Pediatric infectious disease journal,* 24(2), pp. 128-131.

14. TROTTER, C.L., ANDREWS, N.J., KACZMARSKI, E.B., MILLER, E. and RAMSAY, M.E., 2004. Effectiveness of meningococcal serogroup C conjugate vaccine 4 years after introduction. *Lancet,* 364(9431), pp. 365-367.

15. BORROW, R. and FINDLOW, J., 2009. Prevention of meningococcal serogroup C disease by NeisVac-C. *Expert review of vaccines,* 8(3), pp. 265-279

16. JOINT COMMITTEE ON VACCINATION AND IMMUNISATION, July 13, 2013. Meningococcal sub-committe - Minute of the meeting.

17. ADVISORY COMMITTE ON IMMUNIZATION PRACTICES, March 22, 2013. Prevention an Control of Meningococcal Disease.

18. RICHMOND, P., BORROW, R., GOLDBLATT, D., FINDLOW, J., MARTIN, S., MORRIS, R., CARTWRIGHT, K. and MILLER, E., 2001. Ability of 3 different meningococcal C conjugate vaccines to induce immunologic memory after a single dose in UK toddlers. *The Journal of infectious diseases,* 183(1), pp. 160-163.

19. BORJA-TABORA, C., MONTALBAN, C., MEMISH, Z.A., VAN DER WIELEN, M., BIANCO, V., BOUTRIAU, D. and MILLER, J., 2013. Immune response, antibody persistence, and safety of a single dose of the quadrivalent meningococcal serogroups A, C, W-135, and Y tetanus toxoid conjugate vaccine in adolescents and adults: results of an open, randomised, controlled study. *BMC infectious diseases,* 13, pp. 116-2334-13-116.

20. VESIKARI, T., FORSTEN, A., BOUTRIAU, D., BIANCO, V., VAN DER WIELEN, M. and MILLER, J.M., 2012. A randomized study to assess the immunogenicity, antibody persistence and safety of a tetravalent meningococcal serogroups A, C, W-135 and Y tetanus toxoid conjugate vaccine in children aged 2-10 y. *Human vaccines & immunotherapeutics,* 8(12),.

21. FRASCH, C.E., BORROW, R. and DONNELLY, J., 2009. Bactericidal antibody is the immunologic surrogate of protection against meningococcal disease. *Vaccine,* 27 Suppl 2, pp. B112-6.

22. DE VOER, R.M., VAN DER KLIS, F.R., ENGELS, C.W., SCHEPP, R.M., VAN DE KASSTEELE, J., SANDERS, E.A., RIJKERS, G.T. and BERBERS, G.A., 2009. Kinetics of antibody responses after primary immunization with meningococcal serogroup C conjugate vaccine or secondary immunization with either conjugate or polysaccharide vaccine in adults. *Vaccine,* 27(50), pp. 6974-6982.

23. BERNASCONI, N.L., TRAGGIAI, E. and LANZAVECCHIA, A., 2002. Maintenance of serological memory by polyclonal activation of human memory B cells. *Science (New York, N.Y.),* 298(5601), pp. 2199-2202.

24. DE VOER RM., 2010. Meningococcal C specific immune responses. immunity in an era of immunization with conjugate vaccine. *Utrecht: University of Utrecht / RIVM,* .

25. DE VOER RM, SANDERS EA, ENGELS CW, KERKHOF J, RIJKERS GT, BUISMAN AM, ET AL., Cellular immunity after immunization woth meningococcal serogroup C conjugate or polysaccharide vaccine in adults. *Unpublished work,*

26. TROTTER, C.L., FINDLOW, H. and BORROW, R., 2012. Seroprevalence of serum bactericidal antibodies against group W135 and Y meningococci in England in 2009. *Clinical and vaccine immunology : CVI,* 19(2), pp. 219-222.

27. MEERVELD-EGGINK, A., DE WEERDT, O., DE VOER, R.M., BERBERS, G.A., VAN VELZEN-BLAD, H., VLAMINCKX, B.J., BIESMA, D.H. and RIJKERS, G.T., 2011. Impaired antibody response to conjugated meningococcal serogroup C vaccine in asplenic patients. *European journal of clinical microbiology & infectious diseases : official publication of the European Society of Clinical Microbiology,* 30(5), pp. 611-618.

28. DE VOER RM, VAN DER KLIS FR, ENGELS CW, RIJKERS GT, SANDERS EA, BERBERS GA., 2008 Aug. Development of a fluorescent-bead-based multiplex immunoassay to determine immunoglobulin G subclass responses to Neisseria meningitidis serogroup A and C polysaccharides. *Clin Vaccine Immunol. 2008 Aug,* 15(8), pp. 1188-93.

29. VAN GAGELDONK, P.G., VAN SCHAIJK, F.G., VAN DER KLIS, F.R. and BERBERS, G.A., 2008. Development and validation of a multiplex immunoassay for the simultaneous determination of serum antibodies to Bordetella pertussis, diphtheria and tetanus. *Journal of immunological methods*, 335(1-2), pp. 79-89.
